# Plasmonic semi shells derived from simultaneous in situ gold growth and anisotropic acid etching of ZIF-8 for photothermal ablation of metastatic breast tumor

**DOI:** 10.1038/s42004-024-01317-w

**Published:** 2024-10-09

**Authors:** Kritika Sood, Purvi Mathur, Sulagna Rath, Pranjali Yadav, Navneet Kaur, Priyanka Sharma, Mimansa, Deepak Singh Chauhan, Sonalika Vaidya, Rohit Srivastava, Abhijit De, Asifkhan Shanavas

**Affiliations:** 1 Inorganic & Organic Nanomedicine (ION) Lab, Institute of Nano Science and Technology, Sector 81, Knowledge City, Mohali, 140306 Punjab India; 2 Advanced Centre for Treatment Research & Education in Cancer, Tata Memorial Centre, Kharghar, Sector 22, Navi Mumbai, 410210 Maharashtra India; 3 Department of Biosciences and Bioengineering, Indian Institute of Technology Bombay, Powai, Mumbai, 400076 Maharashtra India; 4 Present Address: Department of Microbiology and Immunology, Dalhousie University, Halifax, 6299 NS Canada

**Keywords:** Nanobiotechnology, Nanomedicine

## Abstract

Open nanoshells such as nanobowls or nanocups collectively described as ‘semi shells’ have unique plasmonic properties due to their lack of symmetry. So far, their fabrication was based on multistep and laborious methods such as solid state sputter coating or selective deposition/etching using sacrificial templates. In this work, we report a rapid one step colloidal synthetic protocol for PEGylated semi-shell (SS) fabrication by simultaneous facet specific anisotropic chemical etching of rhombic dodecahedral ZIF-8 and heterogenous nucleation & growth of gold. The SS possesses a strong localized surface plasmon resonance in the near-infrared region, which is retained after surface passivation with polyethylene glycol and subsequent cryopreservation for extended shelf-life. Freshly reconstituted PEGylated SS was found to be safe & non-toxic in healthy C57BL/6 mice post intravenous administration. The PEGylated SS displayed significant photothermal efficiency of ~37% with 808 nm laser irradiation. Preclinical assessment of intra-tumoral photothermal efficacy indicated complete remission of primary breast tumor mass with insignificant metastasis to vital organs in 4T1 FL2 tumor bearing CD1 nude mice. Further, PEGylated SS mediated photothermal therapy also yielded morbidity free survivael of 75% for up to 90 days, indicating their potential to significantly improve outcomes in advanced breast tumors.

## Abstract (web summary)

Anisotropic gold-based colloidal nanoparticles such as semi shells have unique optical properties and widespread applications such as in bioimaging, biosensing and therapy, however, the fabrication process of semi shells remains a challenge due to laborious multistep solid state and colloidal procedures. In this study, the authors develop a rapid one-pot colloidal synthetic route for the fabrication of semi shells using ZIF-8 as a sacrificial template, and demonstrate their promise for photothermal therapy.

## Introduction

Colloidal gold is an excellent nanotransducer that can catalyze non-invasive radiations such as light, radiofrequency, ultrasound, and X-ray into destructive secondary thermal and auger radiations to provide precisely localized therapeutic intervention. Ultrasmall and anisotropic gold-based colloidal nanoparticles have been gaining a lot of attention due to the emergence of unique optical & magnetic properties either in autonomous or amalgamated forms, hence finding widespread applications such as bioimaging, biosensing, and therapy^1–5^. Gold nanostructures have unique optical properties based on their localized surface plasmon resonance which is observed due to collective oscillations of conduction electrons in the valence band of these metal nanoparticles excited by the incident radiation. This resonance spectrum is dependent upon the geometry of these nanoparticles, such as nanorod and nanoshell^6^. The nanoshells are known to generate more heat per nanoparticle due to their overall larger geometric cross-section and are found to impart significant prostate cancer ablation in a clinical set up^7^.

Breaking the symmetry of plasmonic nanoparticles enables anisotropic scattering due to differences in plasmonic modes at different angles. Open nanoshells such as nanobowls or nanocups, collectively described as semi-shells, have unique plasmonic properties due to their lack of symmetry^8^. These semi-shells have two distinct dipole resonances i.e., axial mode (parallel to the axis of symmetry) and transverse mode (perpendicular to the axis of symmetry). The transverse mode provides a strong magnetic component to the plasmon resonance due to a current loop generated by the oscillating electrons and the dielectric medium on the surface of the metallic semi-shells. This hybrid plasmonic resonance, along with the charge accumulation at the edge of the rim, results in a large field enhancement, leading to a red shift in the transverse plasmon resonance mode^9^. The magnetic dipole plasmonic mode also confers light bending character to these semi-shells^10^. The possession of these unique properties by semi-shells warrants their application as metamaterials, SERS enhancers, and phototherapeutic agents. However, the fabrication process of semi-shells remain a challenge due to laborious multistep solid state and colloidal procedures. In continuation to nanoshells, asymmetric semi-shell structures such as nanocaps, nanocups and half shells have been synthesized by various groups^9,11–13^. Such protocols typically involve templates such as a monolayer of silica or polymer-based spherical nanoparticles on a silicon substrate sputter coated with gold, followed by dissolution of the template. Top-down methods such as ion milling or electron beam-induced ablation have also been reported^9,14^. Among colloidal synthetic procedures, gold nanocups and nano bottles from vertex-initiated growth on PbS nano octahedrons have been reported with highly monodispersed shapes and size^15^. On the other hand semi-shell structures have also been derived by thermal dewetting of gold nanoshells sandwiched between the inner core and an outer shell of SiO_2_ using calcination and chemical etching^3^. While several potential colloidal protocols exist for deriving at the semi-shell morphology, a simplified method employing non-toxic reactants at room temperature is highly desired as a greener alternative.

**Scheme. 1:**
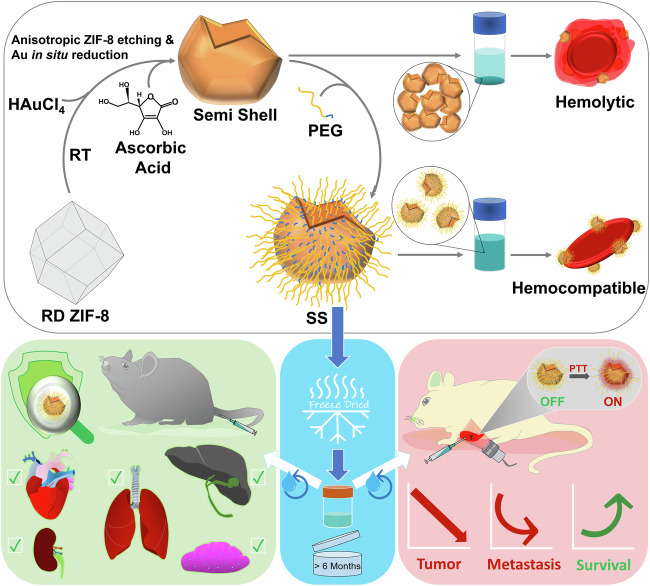
Synthesis and application of plasmonic semi-shells. Illustration of semi-shell formation using ZIF-8 as a sacrificial template and its PEGylation-assisted hemocompatibility, cryopreservation, and on-demand reconstitution towards pronounced photothermal therapy.

In this work, we have developed a rapid colloidal single-step procedure for the formation of semi-shells at room temperature using a biocompatible zinc-based metal-organic framework, ZIF-8 as a sacrificial template. The highly porous nature and anisotropic etching behavior of ZIF-8^16,17^ are found to sequentially enable gold nucleation and its further growth into semi-shells with the simultaneous dissolution of the template. The semi-shells were passivated with polyethylene glycol and also acted as cryoprotectants for lyophilization towards on-demand aqueous reconstitution. The PEGylated semi-shells were found to cause complete ablation of the tumor at the primary site of photothermal treatment and also inhibited pulmonary metastasis with improved survival (Scheme 1).

## Results and discussion

### Synthesis of PEGylated semi-shells (SS)

**Fig. 1:**
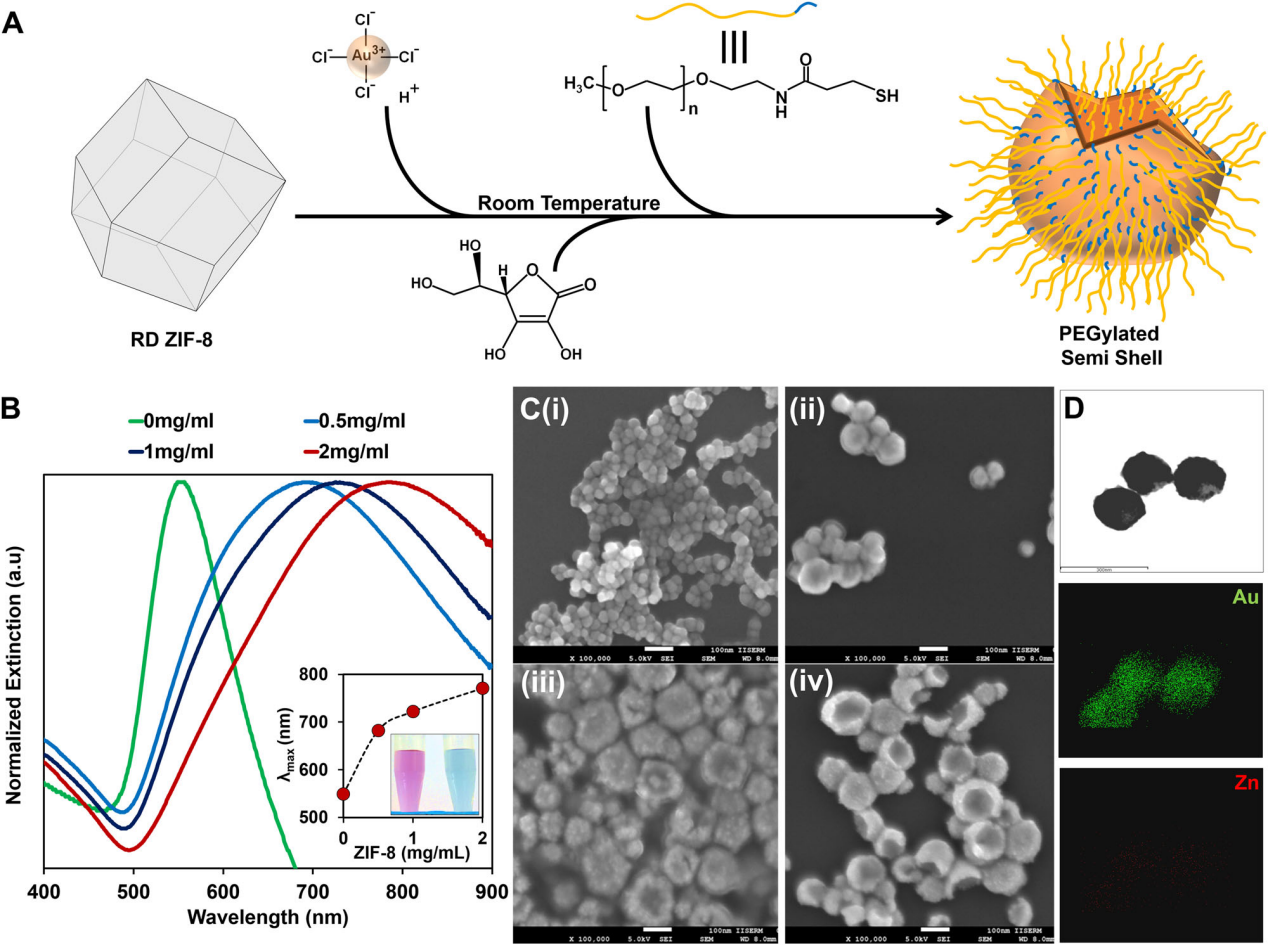
RD-ZIF-8 templated one-pot synthesis of gold semi-shells. **A** Schematic of semi-shell synthesis; **B** Extinction spectra and **C** FESEM images of semi-shells prepared with (i) 0 mg mL^−1^, (ii)−0.5 mg mL^−1^, (iii)−1 mg mL^−1^, (iv)−2 mg mL^−1^ of ZIF-8. Inset in **B** shows a shift in *λ*_max_ and color change of reaction mixture from light ruby red to cyan in the presence of ZIF-8; **D** Elemental mapping of SS for gold and zinc.

Taking advantage of the dissolution behavior of ZIF-8 under acidic conditions, we employed them as sacrificial templates for in situ nucleation and growth of anisotropic gold nanoparticles. The room temperature-driven rapid one-pot synthesis involves a mixture of chloroauric acid and rhombic dodecahedron ZIF-8 followed by a reduction of gold with ascorbic acid (Fig. 1A). ZIF-8 feed concentration plays a vital role in determining the final shape of the gold nanoparticles, which also provided initial insights into the evolution of semi-shell structures. A distinct color difference of light ruby red and cyan was observed between the colloids synthesized in the absence and presence of ZIF-8, respectively. The LSPR spectra of gold nanoparticles derived with different concentrations of ZIF-8 showed a linear red shift in the absorption maxima (Fig. 1B). FESEM analysis confirmed concave discoid shapes at lower concentrations to semi-shells at higher concentrations of ZIF-8, while gold and ascorbic acid molar concentrations remained constant (Fig. 1C). Typical gold nanospheres were formed in the absence of ZIF-8. Due to complete solubilization, ZIF-8 structures were mostly absent in the semi-shell colloid after post-synthetic washing in double distilled water. However, the elemental mapping detected zinc being retained in the semi-shells (Fig. 1D) with Au:Zn weight ratio of 10:1 as quantified with ICP-MS.

The as-prepared semi-shells rapidly precipitated due to their anisotropic shape. Passivating the semi-shell surface with polyethylene glycol significantly improved its colloidal stability. A PEG:Au molar ratio of 0.17 provided optimal grafting of ~82% (Table S1). Hydrodynamic size and zeta potential of PEGylated semi-shells were measured to be ~190 nm and ~ −15 mV (Fig. S1). The size of as-prepared semi-shells could be tuned based on the size of ZIF-8, while agglomeration of the template interestingly leads to the formation of flower petal-like arrangement of the semi-shells (Fig. S2).

### Mechanism of ZIF-8 templated formation of semi-shell

The mechanism of formation of solid semi-shell structures was investigated in conjunction with prior reports on anisotropic acid etching of ZIF-8. ZIF-8 with rhombic dodecahedron (RD) structure possesses six {100} surfaces, which are present at the corners bound by four edges, eight {111} surfaces, present at the corners bound by three edges, and {211} surfaces present on the edge of the RD. Amongst these surfaces, {100} and {211} are known to be prone to etching in acidic environments^17^. For the formation of a hollow semi-shell, we hypothesize that mixing of a strong monoprotic conjugate acid, HAuCl_4_ (5 mM stock pH~2), with RD-ZIF-8 induced simultaneous integration of Au^3+^ at the surface and initiated the formation of Kirkendall voids. Such Kirkendall voids are known to form in ZIF-8, wherein polyhedrons (regular hexahedron; RH and rhombic dodecahedron; RD) of ZIF-8 in the presence of Au^3+^ or Pt^2+^ undergo partial surface substitution of Zn^2+^ forming a bimetallic stable shell followed by complete diffusion of Zn^2+^ from the core. However, contrary to the prior observation of co-aggregation of the guest metal (Au^3+^ or Pt^2+^) and Zn^2+^^18,19^, we noted monocrystalline nanoparticles on the surface of RD-ZIF-8. These nanoparticles were analyzed to be Au, as confirmed by the observed lattice spacing of 0.232 nm corresponding to the Au (111) plane. As ZIF-8 has an established host-guest interaction within its micropores, it is rational to expect Au^3+^ to diffuse into the pore aperture and nucleate inside these pores due to the mild reducing property of the imidazole groups.

**Fig. 2:**
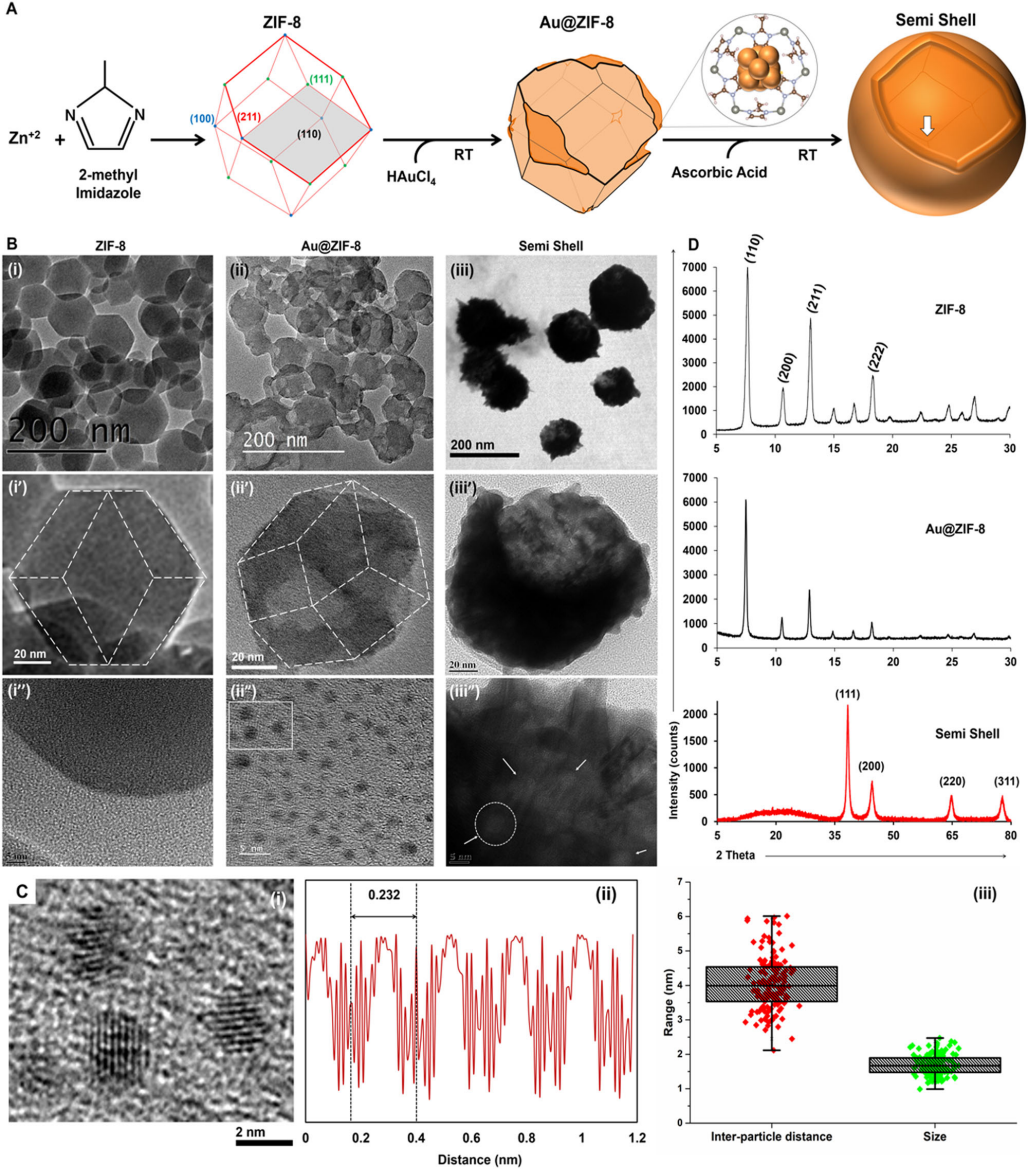
Elucidation of semi-shell formation using TEM and XRD. **A** Summarized mechanism of semi-shell formation from ZIF-8 template; **B** TEM images of ZIF-8 (i, i’ & i”), Au@ZIF-8 (ii, ii’ & ii”) and SS (iii, iii’ & iii”) at different magnifications (white arrows indicate remnant holes formed for outward diffusion of Zn^2+^ from core); **C** High magnification TEM image of insert in B-ii” (i), lattice spacing profile (ii) and size & inter-particle distance (iii) of gold nanoparticles within Au@ZIF-8 (*n* = 150). The arrow in **A** points at the indentation of {111} vertex of the ZIF-8 at the bottom of the semi-shell observed in Fig. 1C (iv); **D** XRD pattern of ZIF-8, Au@ZIF-8 and SS.

X-ray photoelectron spectroscopy of these Au@ZIF8 revealed high-resolution peaks centered at 84.85 eV and 88.9 eV corresponding to Au 4f_7/2_ and Au 4f_5/2_ respectively, for Au(I) and peaks at 84.07 eV and 87.66 eV corresponding to Au(0) (Fig. S3A). The relative ratio of Au(I) to Au(0) was found to be 3:1. This observation indicates a structure similar to gold nanoclusters with Au(0) at the core and Au(I) at the surface interacting with a finite number of ligand, herein 2-methylimidazole. Additionally, peaks centered at 88.24 eV and 91.44 eV corresponding to Zn 3p_3/2_ and 3p_1/2_ for Zn in the +2 oxidation state were observed. The presence of Zn3p is consistent with previous reports for Au-Zn nanocomposites^20,21^. Peaks corresponding to Zn 2p_3/2_ and 2p_1/2_ were also observed at ~1021 eV and ~1044 eV in both ZIF-8 and Au@ZIF-8 (Fig. S3B, C). The uniform and stable distribution of gold nanoparticles with average size of ~1.7 nm and mean inter-particle distance of ~4 nm (Fig. 2C iii) is expected to have formed within the central cavity of the unit cells of the ZIF-8 matrix that may expand in volume to accommodate host entities^22,23^. Prior reports indicate that gold nanoparticles with a wide size range of 1–5 nm can be incorporated into ZIF-8 with local defects & deformities^24^.

In addition to inducing such local defects for accommodating gold nanoparticles within the ZIF-8 framework, the acidic condition brought by HAuCl_4_ also results in the etching of {100} and {211} surfaces of ZIF-8. This anisotropic etching was evident from the XRD analysis of Au@ZIF-8 (Fig. 2D), wherein a reduction in the intensity of (100) and (211) planes by ~25% and ~43%, respectively, was observed relative to the (110) plane. The {110} surface is predominantly stable due to the absence of the acid-prone Zn-2-imidazole linkages [17]. TEM analysis of Au@ZIF-8 (Fig. 2B ii’) also revealed the etching of {100} and {211} surfaces of ZIF-8, which is consistent with the previous report^17^. The addition of ascorbic acid (20 mM stock pH~3) to the Au@ZIF-8 accelerated the degradation of the ZIF-8 framework. This could result in the coalescing of the gold aggregates present on the surface of ZIF-8 to grow into a semi-shell morphology.

Interestingly, as seen with FESEM (Fig. 1C iv) and TEM (Fig. 2B iii & iii’), the majority of the semi-shells displayed a single opening. This is surprising as the symmetric distribution of the (100) and (211) planes is expected to yield at least six independent openings if etched along the six <100> axial directions. However, FESEM (Fig. S4) and TEM (Fig. 2B ii’) analysis of Au@ZIF-8 indicated mostly three clear openings. These three openings are observed to be present on the same side of the hemisphere of RD-ZIF-8. It is presumed that further asymmetrical etching along {100} and {211} surfaces in these three openings, which is expedited by the addition of ascorbic acid, has resulted in the formation of a larger opening (the opening of the semi-shell). This is also confirmed by the presence of an indentation corresponding to the {111} vertex of the ZIF-8 at the bottom of the semi-shell (Fig. 2A). Careful observation of RD-ZIF-8 structure reveals that the {100} surface present at the corner and the {211} surface present on the edges of the RD are not along the same planar level, which could explain the formation of undulated openings of the semi-shell. One could also observe that there are openings symmetrically opposite to each other in Au@ZIF-8 (Fig. S5B) which further confirms our hypothesis.

**Fig. 3:**
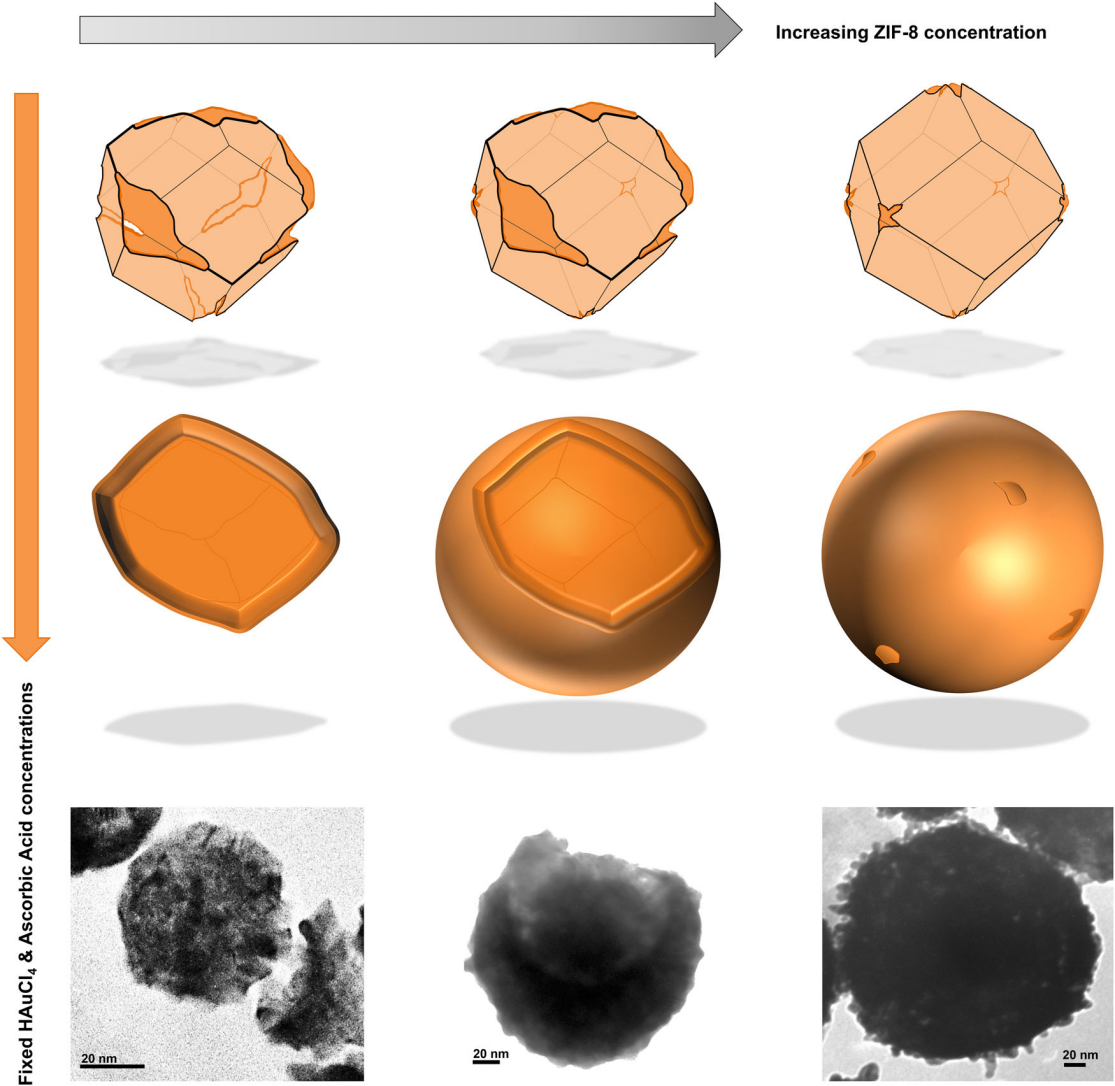
Formation of different semi-shell morphology at varying RD-ZIF-8 concentrations. ZIF-8 concentration-dependent morphological outcomes of gold nanostructures at a constant feed concentration of chloroauric acid and ascorbic acid.

The polycrystallinity, the phase-purity of the semi-shell, and the absence of structurally intact ZIF-8 at the end of the reaction were confirmed with XRD (Fig. 2D). The ZIF-8 concentration-dependent formation of semi-shells with cap and cup shapes could be well correlated with this hypothesis (Fig. 3 and S6). At higher concentrations (>3 mg mL^−1^) of ZIF-8, spherical porous nanoshells were observed. This could be explained by the fact that increasing the amount of ZIF-8 at a constant molar concentration of HAuCl_4_ and ascorbic acid would result in inadequate etching of the {100} and (211} surfaces. The spherical porous nanoshells thus formed at higher concentrations of ZIF-8 did not have any superior orifice and possessed a broader plasmon resonance band (Fig. S6). Additionally, we also studied the formation of semi-shells after adjusting ZIF-8 to pH~10 to neutralize protons contributed by HAuCl_4_. Intriguingly, even after the sequential addition of both HAuCl_4_ and ascorbic acid, the Au@ZIF-8 was predominantly intact, displaying shapes similar to semi-shells, mostly with one large opening (Fig. S7). These structures also clearly showed several holes in the shell involved in the outward diffusion of Zn^2+^ from the core. Remnants of these holes could also be traced within the final semi-shell structures as observed with TEM (Fig. 2B iii”).

### Lyophilization and aqueous reconstitution of PEGylated semi-shells (SS)

**Fig. 4:**
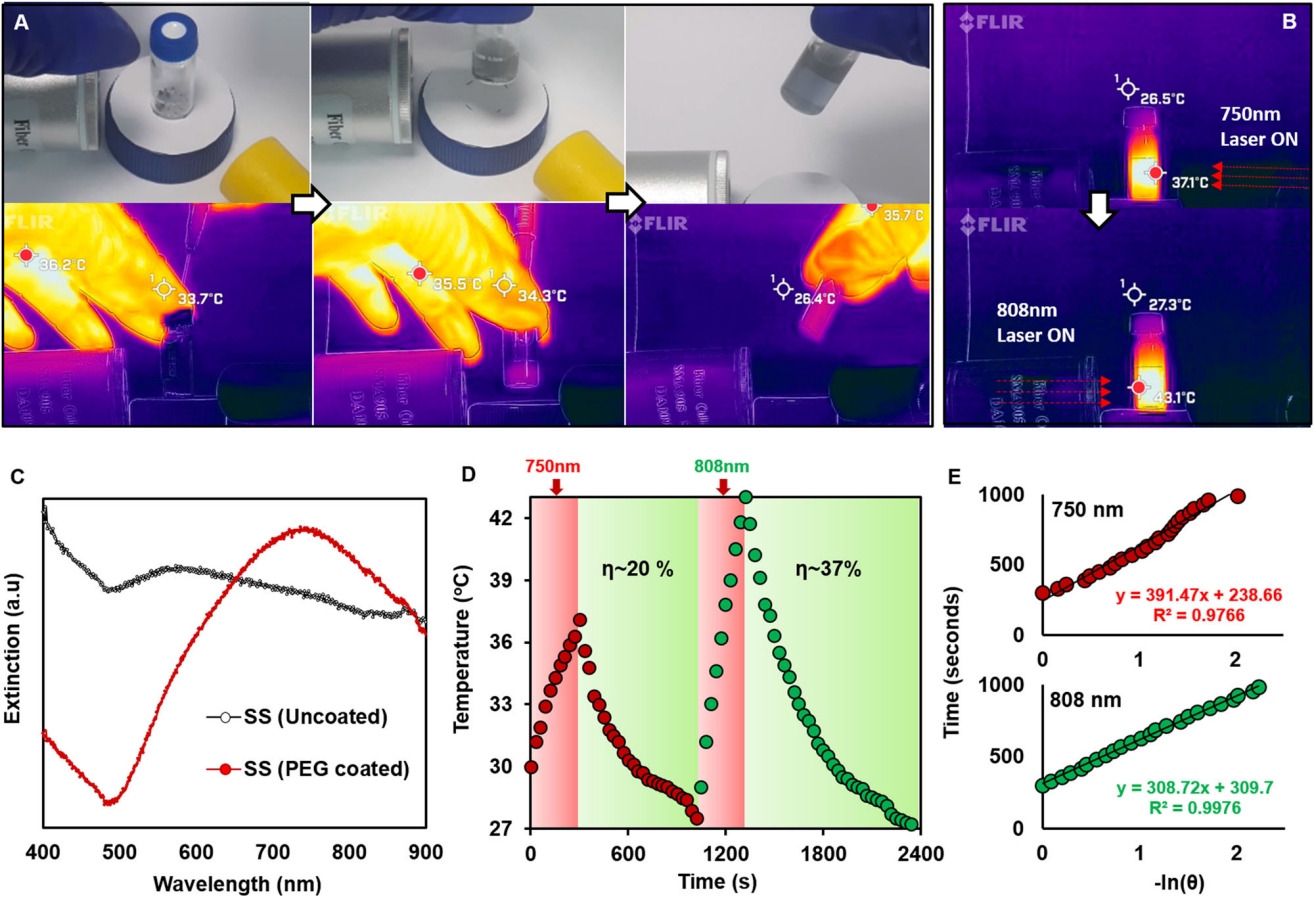
Evaluation of optimal photothermal parameters with freshly reconstituted Lyophilized PEGylated semi-shells. **A** Ambient light (top) and thermal (bottom) images of the reconstitution of SS (1 mg) in 0.9% saline (1 mL); **B** Thermal images of reconstituted SS post irradiation with 750 nm and 808 nm lasers for 300 s; **C** Extinction spectra of semi-shells (uncoated & stored overnight post-synthesis) and freshly reconstituted PEGylated SS; **D** Heating & cooling cycles after sequential irradiation of SS with 750 nm and 808 nm lasers; **E** Plots of linear fitting cooling time versus negative natural logarithm of temperature driving force post laser irradiation with 750 nm (red) and 808 nm (green).

While PEGylation significantly improved the colloidal stability of the semi-shells, long-term storage of SS is plausible only with cryopreservation. PEG has been successfully utilized in the past as both a stabilizer and cryoprotectant of gold nanoparticles^25^. Lyophilized SS could be readily reconstituted in double distilled water or 0.9% (w/v) saline after refrigerated storage for up to 6 months (Fig. 4A and Supplementary video 1). The reconstituted SS showed blue shift in the plasmonic peak, however retained thermogenic property when irradiated with near-infrared light. Sequential irradiation of freshly reconstituted SS with 750 nm and 808 nm CW lasers at fixed power (Fig. 4B–D and Supplementary video 2) and time of 650 mW cm^−2^ and 300 s, respectively, yielded ~20% and ~37% photothermal transduction efficiencies (Fig. 4E and Table-S2). As a prior report shows the influence of zinc doping in gold nanoparticles on their optical response^26^, a dedicated investigation may aid in understanding the effect of different percentages of zinc doping in gold SS on its photothermal efficacy. Further, SS was found to have excellent photothermal stability with up to five cycles of intermittent 808 nm laser irradiation (Fig. S8), confirming its potential as a photothermal nanotransducer.

### In vitro and pre-clinical safety assessment of SS

**Fig. 5:**
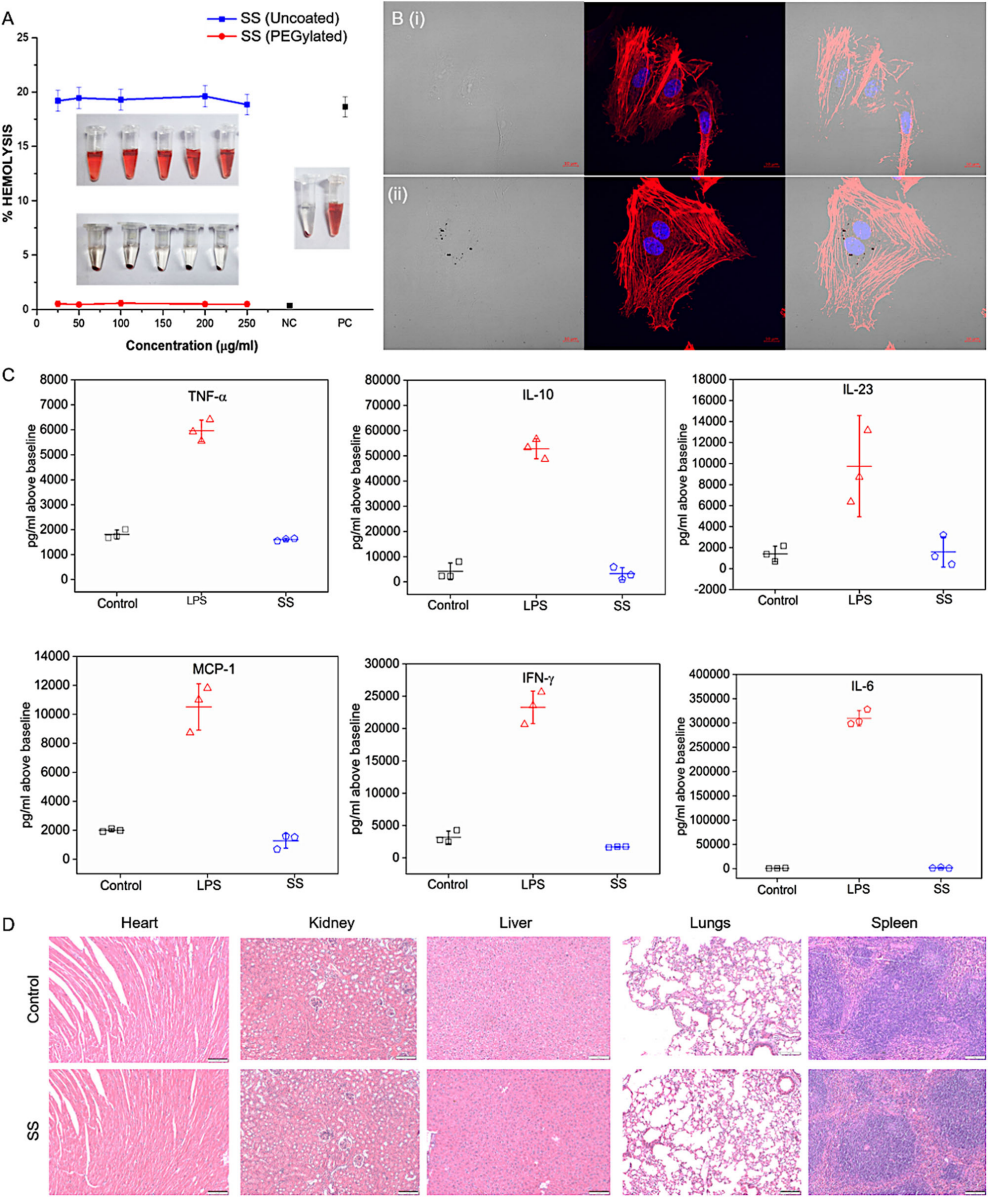
In vitro and pre-clinical safety assessment of SS. **A** Hemolysis assessment of uncoated and PEG-coated semi-shells; **B** HUVEC cells treated with blank media (i) & media containing 50 µg/mL SS (ii) and stained for actin (red) and nucleus (blue) (scale bar: 10 µm); **C** Levels of serum inflammatory markers 24 h post intravenous injection with saline (control), lipopolysaccharide (LPS) and SS; **D** Hematoxylin & Eosin staining of vital organs post treatment at day-1.

As it is imminent for the SS to come in contact with blood post in vivo administration for therapeutic applications, its hemocompatibility was primarily assessed. It was observed that irrespective of the concentration, uncoated semi-shells caused severe hemolysis similar to positive control. However, semi-shells passivated with PEG at concentrations up to 250 µg mL^−1^ did not cause any obvious hemolysis (Fig. 5A). The SS also showed excellent dose-dependent uptake in HUVEC cells with clear distribution within the cytoplasm and possessed fairly good viability of ~79% at 100 µg/mL with well-retained morphology (Fig. 5B and S9). Further, acute (1-day post injection) and sub-acute (28 days post injection) toxicity assessment was performed with intravenous injection of SS (5 mg/Kg equivalent of Au) at the tail vein of healthy C57BL/6 mice. ICP-MS analysis of organs indicated predominant accumulation in the liver and the spleen post 28 days, which is similar to the trend observed for similarly sized gold nanoparticles (Fig. S10A)^27,28^. Serum levels of acute inflammatory cytokines such as MCP-1, TNF-α, IFN-ɤ, IL-6, IL-10, and IL-23 remained normal (Fig. 5C). Body weight of SS injected mice remained the same as that of saline control (Fig. S10B). Serum biomarkers such as aspartate aminotransferase, alanine aminotransferase, blood urea creatinine, and creatinine analyzed post day 28 of injection were found to be within the normal reference range for C57BL/6 mice (Fig. S10C). Histopathological analysis with hematoxylin and eosin staining of vital organs did not indicate any signs of acute or sub-acute tissue damage (Fig. 5D). These observations established pre-clinical safety for further phototherapeutic assessment of SS.

### Photothermal therapy of metastatic breast tumors with SS

**Fig. 6:**
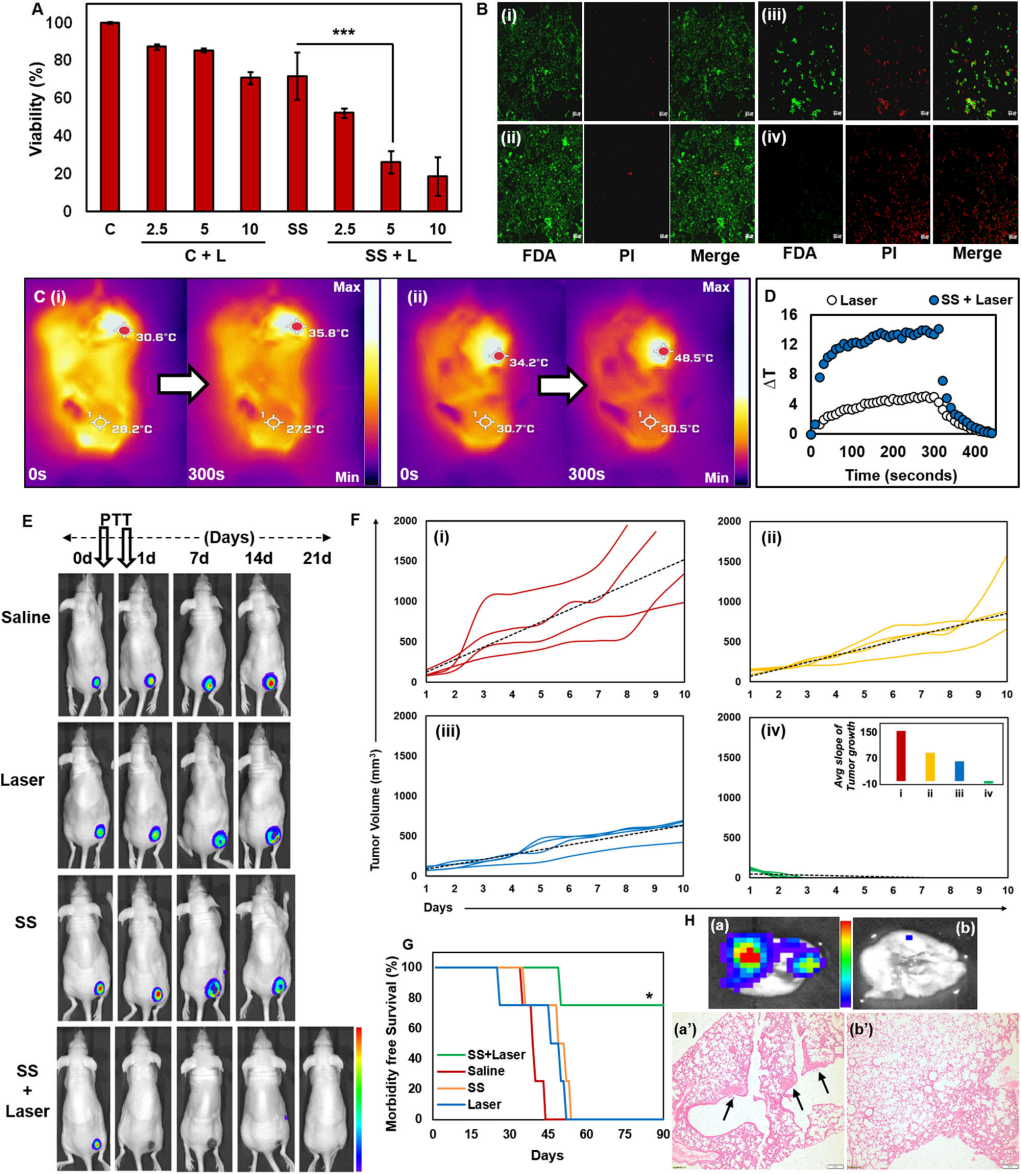
In vitro and pre-clinical photothermal therapeutic evaluation with SS. **A** Irradiation time-dependent photothermal toxicity of SS in MDA-MB 231 cells (***p < 0.001; *n* = 3); **B** Live/dead staining of cells in control (i), 5 min laser alone (ii), SS alone (iii) & SS + 5 min laser (iv) groups (Scale bar: 200 µm); **C** Thermal images of representative mouse in laser (i) & SS + laser (ii) groups; **D** Temperature profile obtained from **C**; **E** Periodic bioluminescence imaging of representative 4T1 FL2 tumor bearing CD1 nude mice in control groups (*n* = 3) and SS + laser group (*n* = 5); **F** Time-dependent change in 4T1 tumor volume in saline (i), laser alone (ii), SS alone (iii) & SS + laser (iv) groups of Balb/c mice (inset in [iv] show tumor growth slopes); **G** Kaplan–Meier survival analysis of mice in control and treatment groups (*n* = 4) (*p < 0.05 for individual control groups *Vs* SS+Laser); **H** Ex vivo bioluminescence imaging of representative lungs in saline (a) and SS + laser (b) groups post autopsy and the micrographs of haematoxylin & eosin stained sections in these two groups post survival analysis (a’ & b’) (black arrows indicate metastatic tumor lesions).

Preliminary assessment of SS for photothermal therapy was performed against triple-negative breast cancer MDA-MB-231 cells at 100 µg mL^−1^ concentration and different 808 nm laser irradiation times. While there was irradiation time-dependent toxicity was observed, 5 min duration was found to be optimal (Fig. 6A). Further, live/dead assay indicated >95% non-viable cancer cells when SS is combined with laser, in comparison to the blank and laser controls with >95% viable cells. Interestingly, material control (SS alone) caused cellular clumping with >30% of dead cells (Fig. 6B). As this extent of toxicity was not observed at the same concentration in highly sensitive normal HUVEC cells, the cancer-specific toxicity could be due to the glutathione depleting effect of gold nanoparticles^29^. Proceeding forward for evaluation in pre-clinical tumor models, the ability of SS for on-demand reconstitution in saline for in vivo administration and photothermal ablation was confirmed (Supplementary video 3). The combination of SS and 808 nm laser irradiation provided ~14 ^o^C rise in temperature as compared to only ~5 ^o^C with laser control (Fig. 6C, D and Supplementary video 4). The achieved temperature rise is well within the hyperthermic window for irreversible tissue damage associated with microvascular thrombosis, ischemia, and hypoxia^30^.

The phototherapeutic effect of SS was evaluated in 4T1 FL2 tumor-bearing CD1 nude mice for up to 21 days. The mice subjected to one dose of intra-tumoral injection of SS followed by two independent laser irradiations at day-0 and day-1 showed a drastic drop in the tumor bioluminescence signal within the first 24 h (Fig. 6E). However, the control groups continued to show an increase in the tumor growth as assessed by the intensity of bioluminescence signal (Fig. S11). While the photothermal treatment group showed complete ablation of the primary tumor, the control groups were sacrificed at day-14 due to their tumor burden. There was no obvious recurrence in the bioluminescence at the primary inoculated site on the mice within the phototherapeutic group for up to 21 days. While peripheral bioluminescence was observed near the inoculated site, possibly arising from a sentinel lymph node, the mice were noticed to be free from spontaneous metastatic lesions in the lung (Fig. 6H a&b and Fig. S12). Contrastingly, the control groups showed severe metastasis to both lung and chest bones. The average growth of 4T1 tumor over 10 days post treatment indicated steeper and positive slopes in control groups corresponding to rapid proliferation (Fig. 6F i–iii). Nevertheless, the photothermal therapy group showed a deceleration in tumor growth, as observed from the negative slope (Fig. 6F iv). Subsequently, this complete ablation of the primary tumor significantly improved the survival of the mice, with 75% of mice persisting up to 90 days post treatment without any recurrence event (Fig. 6G and S14). Post 90 days, histopathological analysis with hematoxylin & eosin staining of representative lung sections from the saline group indicated several metastatic lesions within the pulmonary alveolar space and tumor cell aggregation in the parenchyma in addition to thickening of the pleural lining. However, the obliteration of the primary tumor in the PTT group suppressed the spread of tumor cells to the lung, as seen from its normal morphology (Fig. 6H a’& b’ and S15).

## Conclusion

We have developed a rapid one-pot colloidal synthetic route for the fabrication of semi-shells (SS) using ZIF-8 as a sacrificial template. The flexible micropores of ZIF-8 and its unique ability to undergo anisotropic etching at acidic pH allowed the nucleation of gold and the formation of the semi-shell morphology. The morphology-dependent emergence of the LSPR band in the NIR region activated the photothermal activity of SS under 808 nm laser irradiation with notable transduction efficiency. Their colloidal stability was improved by functionalizing with PEG which also acted as cryoprotectant for freeze drying towards enhancing the shelf-life of the SS with uncompromised photothermal stability. The SS-mediated photothermal therapy has not only resulted in the complete eradication of the primary tumor but also significantly inhibited its metastasis to vital organs with improved survival. As the method reported in this work allows SS synthesis with minimal environmentally friendly resources, it can be fabricated with ease and might find further application in the fields of sensing and catalysis.

### Materials and methods

Chloroauric acid (HAuCl_4_), 2-Methylimidazole (2-MIM), DTNB (5,5’-dithio-bis-(2-nitrobenzoic acid) and O-[2-(3-Mercaptopropionylamino) ethyl]-O′-methyl polyethylene glycol (M.W:5000 Da) were obtained from Sigma Aldrich. Zinc nitrate hexahydrate and L-ascorbic acid were obtained from TCI chemicals. Methanol was obtained from SDFCL. The glassware used for synthesis was cleaned with aqua regia carefully, rinsed with water thoroughly, and oven dried properly before use. Double distilled water was used throughout the experiments unless mentioned otherwise. All chemicals were of analytical grade and were used as received without any further purification.

### Characterization

UV-visible absorption spectra were recorded at room temperature using a Shimadzu UV-2600 spectrophotometer using a quartz cuvette. Dynamic light scattering (DLS) and Zeta Potential measurements were performed on a Malvern zetasizer (model: nano ZSP). TEM images were recorded using JEOL 2100 with lanthanum hexaboride (LaB6) filament at an accelerating voltage of 200 kV. SEM analysis was done using JEOL JSM IT300. Average nanoparticle size from electron micrographs was measured using Gatan microscopy suite software. XRD measurements were recorded using a Powder X-Ray Diffractometer (Model-D8 Advance) with Cu Kα (*λ* = 1.5406 Ǻ) radiation in the 2*θ* range of 5°–80°.

### Synthesis of a rhombic dodecahedron (RD) ZIF-8

Zinc nitrate hexahydrate (150 mg) and 2-methylimidazole (350 mg) were dissolved in 7.2 mL methanol separately^31^. The 2-methylimidazole solution was added to the zinc nitrate solution dropwise under stirring at 2000 rpm. Post 15 min of stirring, the solution was washed with methanol thrice while centrifuging at 7500 rpm for 15 mins. Post washing, the pellet obtained was dissolved in 1 mL double distilled water and sonicated in a water bath until ZIF-8 was properly dispersed and used immediately for semi-shell preparation. It is important to note that ZIF-8 stored in an aqueous medium for the long term undergoes hydrolytic etching and thus might be unsuitable for semi-shell synthesis.

### Synthesis of semi-shells

Typical synthesis involves 400 µL of 2 mg mL^−1^ RD-ZIF-8 stock in water aliquoted in a microfuge tube further made up to 850 µL with water. Next, aqueous HAuCl_4_ solution (5 mM, 150 µL) was added to the ZIF-8 solution, followed by the addition of freshly prepared ascorbic acid solution (20 mM, 175 µL). The color of the mixture instantly turned to cyan, indicating the formation of the semi-shells. The reaction was carried out at room temperature. Only double distilled water (18 MΩ cm^−1^) was utilized.

### Quantification of PEG drafted onto semi-shells via the Ellman’s Assay

PEGylation provides steric and thermodynamic stabilization by controlling intrinsic colloidal aggregation behavior and non-specific protein adsorption to semi-shells in biological media. We investigated the grafting efficiency for different PEG density coated SS using Ellman’s assay^32^.

Briefly, freshly prepared semi-shells were taken at a concentration of 5.072 × 10^15^ particles and were incubated with Thiol-PEG at different working concentrations (1.7 mM, 0.85 mM, and 0.425 mM) overnight. The PEG:Au molar ratios were calculated as 0.34, 0.17, and 0.085, respectively, for these PEG densities. The resultant PEG-modified SS were designated as: PEG-SS_0.34_, PEG-SS _0.17_, and PEG-SS_0.085_. Next day, the PEGylated semi-shells (abbreviated as SS hereafter) were centrifuge washed at 8000 rpm for 30 min, and the supernatant solution was used to detect unreacted thiols.

Briefly, 250 µL of supernatant solution was mixed with 95 µL of Phosphate buffer (0.1 M PB buffer mixed with 1 mM EDTA, pH 8) and 5 µL of 3 mM DTNB. The solutions were mixed thoroughly and were allowed to incubate at room temperature for 15 min^33^. DTNB^−2^ reacts with free sulphydryl groups to yield a mixed disulfide and 2-nitro-5-thiobenzoic acid TNB^−2^ which can be quantified via a colorimetric assay with maximum absorbance at 412 nm. Each sample was prepared in triplicates, and the percentage of reacted thiol was calculated using the formula:

$$\% {{\rm{Reacted}}}\; {{\rm{PEG}}}=\frac{\left({{\rm{Total}}}\; {{\rm{PEG}}}\; {{\rm{added}}}-{{\rm{Free}}}\; {{\rm{PEG}}}\right)\times 100}{{{\rm{Total}}}\; {{\rm{PEG}}}\; {{\rm{added}}}}$$

### Lyophilisation and reconstitution of SS

Post PEGylation, the SS were frozen in liquid nitrogen and subjected to lyophilisation (Operon, Korea; Temp: 120 °C, Pressure: 0.02 bar). The lyophilized SS powder was stored in −20 degrees and reconstituted in water as required. Post reconstitution, UV–Vis spectroscopy and TEM analysis were performed to assess retention of the morphology and optical property.

### Photothermal transduction efficiency of SS

The photothermal transduction efficiency was carried out for SS under sequential irradiation with continuous-wave lasers: 750 nm and 808 nm, in accordance with the previous study by Ropar et al.^34^ and calculated according as follows

The total energy balance for the system,

1 $${\sum}_{i}{m}_{i}{C}_{p,i}\frac{{dT}}{{dt}}={Q}_{{NS}}+{Q}_{{Dis}}-{Q}_{{Surr}}$$

where *m* and *C*_*p*_ are the mass and heat capacity of water respectively, *T* is the solution temperature, *Q*_*NS*_ is the energy input by nanoparticle system. *Q*_*Dis*_ is the baseline energy input by the sample cell, and *Q*_*Surr*_ is heat conduction away from the system surface by air.

2 $${Q}_{{NS}}=I\left(1-{10}^{-A}\right){{\rm{\eta }}}$$

where *I* is incident laser power, *ƞ* is the conversion efficiency from incident laser energy to thermal energy, and *A* is the absorbance of the nanoparticle system at a given laser wavelength (nm).

3 $${Q}_{{Surr}}={hS}\left(T-{T}_{{Surr}}\right)$$

where *h* is the heat transfer coefficient, *S* is the surface area of the container, *T* is the temperature, and *T*_*Surr*_ is the ambient temperature of the surroundings.

At a defined power of laser, ${Q}_{{NS}}+{Q}_{{Dis}}$ is finite. Since the heat output *Q*_*Surr*_ would continue to increase with a rise in temperature *T*, thus, for achieving a maximum rise in temperature, heat input would be equal to heat output:

4 $${Q}_{{NS}}+{Q}_{{Dis}}={Q}_{{Surr}-{Max}}={hS}\left({T}_{{Max}}-{T}_{{Surr}}\right)$$

where *Q*_*Surr-Max*_ is heat conduction away from the system when the sample cell reaches the equilibrium temperature, *T*_Max_ is the equilibrium temperature. The PTT can be obtained by substituting (2) into (4), giving rise to the following equation:

5 $$\eta =\frac{\left[{hS}\left(T\max -{Tsurr}\right)-{QDis}\right]}{I\left(1-{10}^{-A750{or}808}\right)}$$

wherein *T*_Max_ is the steady-state maximum temperature attained by the nanoparticle system, *T*_Surr_ is the ambient room temperature, *Q*_Dis_ is the energy input based on the heat generated by the solvent (water) and samples well or baseline energy input, *I* is the laser power, and A750/A808 is the absorbance of nanoparticles at 750 nm and 808 nm.

To determine *hS* for Eq. 5, a dimensionless driving force temperature, *θ*, is introduced using the maximum system temperature, *T*_max._

6 $$\theta =\frac{T-{T}_{{Surr}}}{{T}_{{Max}}-{T}_{{Surr}}}$$

And a sample system time constant *τ*_s_

7 $${\tau }_{s}=\frac{{\sum}_{i}{{mc}}_{i}{C}_{p,i}}{{hS}}$$

which is substituted into Eq. (1) and rearranged to yield

8 $$\frac{d\theta }{{dt}}=\frac{1}{{\tau }_{s}}\left[\frac{{Q}_{{NS}}+{Q}_{{Dis}}}{{hS}({T}_{{Max}}-{T}_{{Surr}})}-\theta \right]$$

When the laser source was turned off at the cooling stage of the nanoparticle system, *Q*_NS_ + *Q*_Dis_ = 0, thereby reducing the Eq. (8) to

9 $$\frac{d\theta }{{dt}}=\frac{-\theta }{{\tau }_{s}}$$

and after integration, giving the expression

10 $$t=-{\tau }_{s}{\mathrm{ln}}\theta$$

The time constant (*τ*_s_) was determined by plotting the time versus the negative logarithm of temperature in the cooling period. Here, *m* is 1 g, and *C* is 4.2 J g^−1^. In addition, the energy input is based on the amount of heat generated by water in the sample well or the baseline energy input (*Q*_*Di*s_ = 1.4 mW and 2.8 mW for 750 nm and 808 nm lasers, respectively). The laser power was (*I* = 650 mW cm^−2^), and the A (absorbance at 750 nm, 808 nm) was substituted in Eq. (5).

Experimental condition for PCE involved reconstitution of 1 mg mL^−1^ of SS in a glass vial and sequentially irradiated with 750 nm and 808 nm laser for 5 min each at a power density of 650 mW cm^−2^. Both the heating and cooling of the samples were measured using a FLIR Pro thermal imaging camera.

### Hemocompatibility of semi-shell

Hemolysis assay was performed using human blood collected from a healthy volunteer with informed consent after obtaining necessary approvals from the institute ethics committee of IIT Bombay (IITB-IEC/2019/031). Briefly, PEG-coated SS and uncoated SS were re-dispersed in PBS and tested for hemolysis at concentrations of 25, 50, 100, 200, and 250 µg mL^−1^. Briefly, 150 µL of RBC fraction was added to 750 µL of PEG-coated and uncoated SS solution at different concentrations, and the mixture was incubated for 1 h at 37 °C in an incubator shaker at 180 rpm. After incubation, the mixture was gently centrifuged, and the supernatant was analyzed for leaked hemoglobin. The absorbance of released hemoglobin was recorded at 577 nm for each sample and subtracted from the reference wavelength recorded at 655 nm using a multimode plate reader. Double distilled water and PBS were used as positive (PC) and negative controls (NC), respectively. The experiment was carried out in triplicates^35^. The percentage hemolysis was calculated using the formula:

$$\% {{\rm{Hemolysis}}}\; {{\rm{Control}}}=\frac{{{\rm{Sample}}}\; {{\rm{absorbance}}}{{-}}{{\rm{NC}}}\; {{\rm{absorbance}}}\times 100}{{{\rm{PC}}}\; {{\rm{absorbance}}}{{-}}{{\rm{NC}}}\; {{\rm{absorbance}}}}$$

### Cell culture

MTT [3-(4,5-Dimethylthiazol-2-yl)-2,5-Diphenyltetrazolium Bromide] was purchased from HiMedia. Phalloidin-tetramethylrhodamine B isothiocyanate (Phalloidin-TRITC) and bisBenzimide H 33342 trihydrochloride (Hoechst 33342) were purchased from Sigma Aldrich. MDA-MB 231 (Triple negative human mammary epithelial adenocarcinoma) and 4T1 (Triple negative mammary epithelial carcinoma cell line from BALB/c mouse) were procured from the National Center of Cell Science (NCCS, Pune, India) and cultured in DMEM supplemented with 10% fetal bovine serum (FBS) and 1% antibiotic antimycotic solution at 37 °C in a humidified incubator containing 5% CO_2_. HUVEC were purchased from Lonza (Cat. No. 2517 A) and maintained in endothelial growth medium supplemented with Bullet kit (Lonza Cat. No. 3162). All the cells were maintained by subculturing twice every week.

### In vitro biocompatibility

The biocompatibility of SS was evaluated using HUVEC cells via MTT assay. HUVEC cells were seeded at a density of 2.5 × 10^4^ cells in a 96-well plate for 24 h at 37 °C and 5% CO_2_ in a total volume of 100 µL. Next day, the media was replaced with different concentrations of SS (0, 25, 50, 100 µg mL^−1^) and incubated for 24 h. The following day, the media containing nanoparticles were removed, and the cells were washed with PBS to remove traces of the sample. Next, 10 µL MTT (5 mg mL^−1^ dissolved in PBS) diluted with 90 µL media was added and incubated for 4 h. After incubation, the media was carefully removed, and 100 µL of DMSO was added to each well and aspirated properly to dissolve the purple formazan crystals. The absorbance was recorded at 595 nm on a multimode plate reader. Cell viability percentage was calculated using the formula:

$${{\rm{Cell}}}\; {{\rm{viability}}} \% =\frac{{{\rm{Absorbance}}}\; {{\rm{of}}}\; {{\rm{sample}}}\; {{\rm{at}}}\, 595{{\rm{nm}}}\times 100}{{{\rm{Absorbance}}}\; {{\rm{of}}}\; {{\rm{control}}}\; {{\rm{at}}}\, 595{{\rm{nm}}}}$$

### Cellular uptake of SS in HUVEC

To study the morphological changes of SS treated HUVEC cells, the bright field images were taken along with confocal images. For confocal analysis, the HUVEC cells were seeded on a coverslip at a density of 5 × 10^4^ cells per well in a 6-well plate for 24 h. Next, SS was added at different concentrations. Post 24 h incubation, the media, along with containing nanoparticles, were removed the following day. The cells were then washed with 1x PBS thrice to remove any trace amounts of sample left. Next, the cells were fixed with 4% paraformaldehyde for about 15 min. The nuclei of fixed cells were stained with Hoechst 33342 for 10 min followed by staining F-actin with Phalloidin-TRITC for 20 min. The cells were mounted on a glass slide and observed with LSM 880 Confocal microscope (Carl Zeiss AG, Germany).

### Time dependant photothermal ablation of cancer cells

To study the effect of laser timing on the photothermal ablation of breast cancer cells, MDA-MB 231 cells in the addition of SS were irradiated for differential time periods (2.5, 5, 10 min) at a fixed concentration of 100 µg/mL using 808 nm laser and the cytotoxicity was evaluated via MTT assay. The experiment was carried out in triplicates and the results were recorded as Cell viability (% Control).

### Live/dead assay

Briefly, 8000 cells were seeded in a 48-well plate and grown till confluency for 24 h. Next day, the SS mixed with fresh media was added at a concentration of 100 µg mL^−1^. The controls, along with the sample, were treated with an 808 nm laser for 10 min. Next day, the media was removed, and the cells were washed 1x with PBS. Then the cells were stained with FDA and PI and incubated at room temperature for 15 min. Next, the staining solution was removed and washed with PBS, followed by immediate imaging with the confocal microscope.

### In vivo biodistribution and acute/sub-acute safety assessment

All experimentation involving animals was performed in accordance with the IISER Mohali (Approval no: IISERM/SAFE/PRT/2021/023). Throughout the study, the mice were housed at animal houses at IISER Mohali in individually ventilated cages at a temperature of 22 ± 2 °C and relative humidity of 50–60% under a 12 h light and dark cycle. For the biodistribution study, 6 weeks old C57BL/6 male mice were divided into three groups, each administered with saline (control), liposaccharide (LPS), and semi-shell (SS) (*n* = 3 per group) and were used for studying safety and biodistribution following a repeated dosage at Day 0 and Day 14 over a course of 28 days. SS dosage was 5 mg kg^−1^ (Au equivalent) through the tail vein. Lipopolysaccharide dosage was 2 mg kg^−1^ only at Day 0 for serving as positive control to acute inflammatory analysis. At endpoints, the blood was collected, and major vital organs- heart, lungs, kidneys, liver, and spleen were harvested. The collected blood was allowed to clot for 30 min at room temperature and was centrifuged at 4000 rpm for 10 min at 4 °C. The serum was collected as the supernatant and analyzed for markers related to inflammation and organ function. The acute inflammatory analysis was carried out on selected markers using the BioLegend LEGENDplex^TM^ Mouse inflammation Panel kit using Flow Cytometry (BD FACSAria Fusion). For organ-specific biomarkers: Serum glutamic oxaloacetic transaminase (SGOT)/aspartate aminotransferase (AST), Serum glutamic pyruvic transaminase (SGPT)/alanine aminotransferase (ALT), Blood Urea Nitrogen (BUN) and Creatinine were assessed for hepatic and renal functioning. To determine the amount of gold present in the major organs post 28 days, ICP-MS analysis was carried out. The harvested organs were weighed and digested with ICP grade HNO_3_ and further diluted with double distilled water prior to elemental analysis. The standard preparation for ionic gold was carried out in the range of 10–500 ppb. For histopathological evaluation, the organs were fixed in 10% formalin immediately after harvesting and processed for hematoxylin and eosin (H&E) staining. The stained slides were then studied under an optical microscope for any microarchitectural changes.

### Pre-clinical bedside reconstitution of SS for intra-tumoral administration, monitoring of photothermal temperature rise, and survival analysis post photothermal therapy

A syngeneic tumor mouse model for human breast cancer was developed using the highly metastatic 4T1 breast cancer cells in 6-week-old female Balb/c mice by injecting 1 × 10^6^ cells per mice dispersed in serum-free media on the right flank of the mice. The mice were observed daily, and the tumor size was measured using a Vernier calliper until a palpable size of 100–120 mm^3^ was developed. The mice were divided into four groups: saline, Laser, SS, and SS+Laser, with *n* = 4 in each group. For intra-tumoral injection, SS powder (0.2 mg) was reconstituted in 100 µL of 0.9% saline, and subsequently, 50 µL was injected directly into the 4T1 tumor grafted on the left flank of female BALB/c mice in the SS groups (SS, SS + L). The saline and Laser groups were intratumorally injected with 0.9% saline. An 808 nm laser was used at 650 mW cm^−2^ power to irradiate the tumor region for 5 min on day 0 and day 1. The body weight and tumor volume of all the animals were periodically recorded until the day of morbidity-related death of mice in the four groups for a period of 3 months. Post death, vital organs in saline (including tumor) and SS+Laser groups were excised for histopathology analysis. The bedside reconstitution of SS and administration, followed by photothermal temperature monitoring of both the head and tumor of the mice were recorded simultaneously with a FLIR Pro thermal imaging camera.

### Bioluminescence imaging of primary tumor and its pulmonary metastasis post photothermal therapy with SS

Preclinical photothermal efficacy for tumor regression analysis and bioluminescence imaging was carried out at ACTREC, Mumbai with prior Institutional Animal Ethics Committee (IAEC) approval (No. 29/2021). The photothermal efficacy of SS was carried out in 4T1 xenograft breast tumor model by subcutaneously implanting 1 × 10^6^ cells 4T1 FL2 cells on the right flank of CD1 Nude mice. Post injecting the cells, the tumor growth was continuously monitored by bioluminescence imaging using IVIS Lumina II imaging system (Caliper Life Sciences, USA) by intraperitoneally administering D-luciferin as substrate (100 μL of 30 mg mL^−1^ per mouse). The imaging was carried out at intervals of 1 min between two subsequent images till the maximum bioluminescence signal was observed. Once the desired tumor volume was achieved (80–100 mm^3^), mice were randomly segregated into different groups: control (Saline), material control (SS alone), laser control (808 nm laser irradiation alone), and treatment (SS+laser) groups with sample size *n* = 3 for control groups and *n* = 5 for treatment group. For periodic monitoring of bioluminescence, laser irradiation of the tumors was performed on Day 0 post-imaging (first laser irradiation) and Day 1 pre-imaging (second laser irradiation). The imaging was continued for all groups at regular intervals in order to assess the efficacy of SS for photothermal therapy. The luminescence signal output was quantified in terms of average radiance (p s^−1^ cm^−2^ sr^−1^) using Living Image v4.4 software and represented by a false color scale as a function of the photons captured by the detector. After sacrificing the mice, the bioluminescence was recorded in order to assess organ metastasis in different groups. Post sacrifice, the serum was collected and stored at −80 °C to carry out serum biochemistry analysis.

### Statistical analysis

Statistical significance was determined using one-way ANOVA (analysis of variance) and Student’s *t*-test.

## Supplementary information

Supplementary Information

Description of Additional Supplementary Files

Supplementary Data

Supplementary Movie 1

Supplementary Movie 2

Supplementary Movie 3

Supplementary Movie 4

reporting-summary

## Data Availability

The authors declare that all source data supporting the findings reported in the main manuscript are provided as supplementary data. The data reported in the supplementary information, including XPS high-resolution spectra of Au-ZIF-8. (Supplementary Fig. 3), Extinction spectra of nanocaps and nanoshell prepared from 0.5 mg mL^−1^ & 3 mg mL^−1^ ZIF-8 (Supplementary Fig. 6), Photothermal stability of reconstituted PEGylated SS (Supplementary Fig. 8), Percentage viability of HUVEC cells post treatment with PEGylated SS (Supplementary Fig. 9), In vivo biodistribution, Body weight and Serum biochemical analyses post intravenous injection of PEGylated SS in C57BL/6 mice (Supplementary Fig. 10), Change in bioluminescence signal of 4T1 FL2 tumor grafted on CD1 nude mice post treatment (Supplementary Fig. 11), Serum biochemical analyses post tumor regression analysis in 4T1 FL2 grafted CD1 nude mice (Supplementary Fig. 13), are available on reasonable request from the corresponding author.
