# Supplementary material for: Plasmonic semi shells derived from simultaneous in situ gold growth and anisotropic acid etching of ZIF-8 for photothermal ablation of metastatic breast tumor
Source: Commun Chem. 2024 Oct 9;7:231. doi: 10.1038/s42004-024-01317-w (PMC11464763; doi:10.1038/s42004-024-01317-w)
Supplement: Supplementary file 1 — Supplementary Information [file 42004_2024_1317_MOESM1_ESM.pdf]

# Plasmonic semi shells derived from simultaneous in situ gold growth and anisotropic acid etching of ZIF-8 for photothermal ablation of metastatic breast tumor

Kritika Sood[1], Purvi Mathur[1], Sulagna Rath[2], Pranjali Yadav[1], Navneet Kaur[1], Priyanka Sharma[1], Mimansa[1], Deepak Singh Chauhan[3#], Sonalika Vaidya[1], Rohit Srivastava[3], Abhijit De[2*], Asifkhan Shanavas[1*]

[1]Institute of Nano Science and Technology, Sector 81, Knowledge City, Mohali 140306, Punjab, India
[2] Advanced Centre for Treatment Research & Education in Cancer, Tata Memorial Centre, Kharghar, Sector 22, Navi Mumbai 410210, Maharashtra, India
[3] Department of Biosciences and Bioengineering, Indian Institute of Technology Bombay, Powai, Mumbai 400076, Maharashtra, India
[#] Present Address: Dalhousie University, Halifax, NS, Canada, Department of Microbiology and Immunology, Halifax, 6299, CA

*Corresponding authors: asifkhan@inst.ac.in and ade@actrec.gov.in

**Table S1: PEG grafting efficiency for different PEG densities on semi shells**

| PEG added/Batch of semi shells | Thiol Added (µM) | PEG: Au Molar Ratio | Free thiol (µM) | Reacted thiol (µM) | Reacted thiol % |
|---|---|---|---|---|---|
| **10 mg/batch** | 1702.2 | 0.34 | 452.88± 10.86 | 1249.31 ±10.86 | 73.39± 0.63 |
| **5 mg/batch** | 851.1 | 0.17 | 150.22± 3.62 | 700.87 ±3.62 | 82.34± 0.42 |
| **2.5 mg/batch** | 425.55 | 0.085 | 98.66± 10.42 | 326.88 ±10.42 | 76.81± 2.44 |

PEG-$SS_{0.34}$ depicted lowest grafting efficiency while the highest % grafting was recorded in PEG-$SS_{0.17}$ at approx. 82% and PEG-$SS_{0.42}$ possessed intermediate grafting efficiency.

**Table S2: Photothermal transduction efficiency**

| Laser | Absorbance of sample | hS (mW/°C) | $T_{max}$ -$T_{surr}$ (°C) | Tau (ζ) (sec) | η (%) |
|---|---|---|---|---|---|
| 750 nm | 0.898 | 10.72 | 10.6 | 391.47 | 19.78 |
| 808 nm | 0.895 | 13.60 | 15.7 | 308.72 | 37.16 |

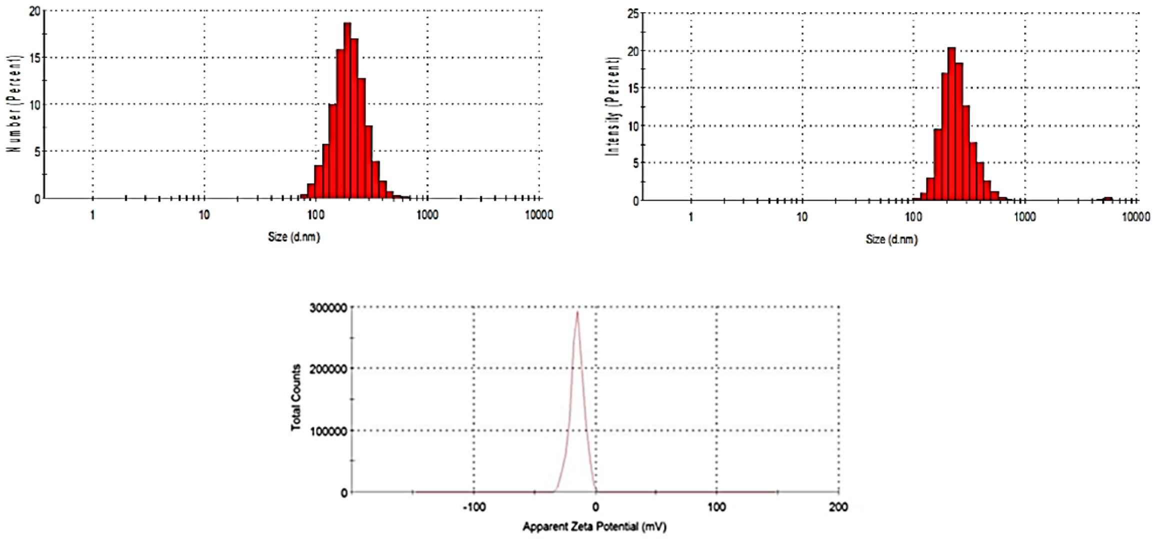


**Figure-S1: Size and charge distribution of reconstituted PEGylated SS**

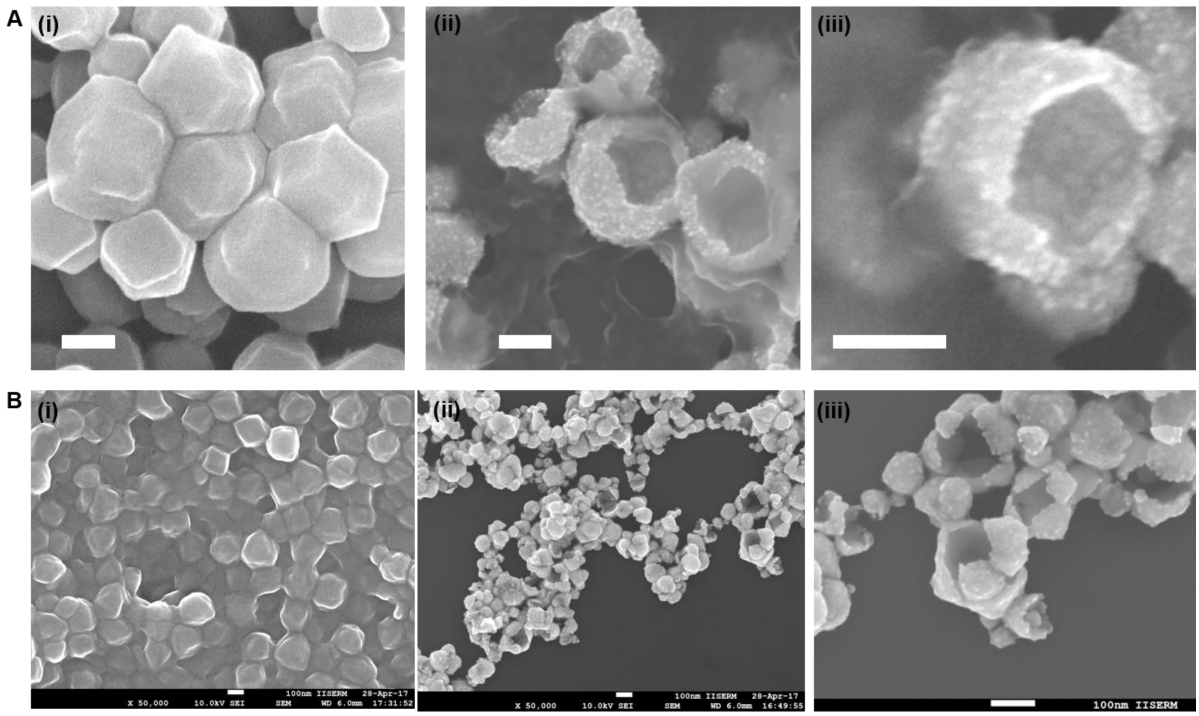


**Figure-S2: Effect of size (A) and agglomeration (B) of ZIF-8 on the morphology of gold nanoparticles. (Scale bar in A – 100 nm)**

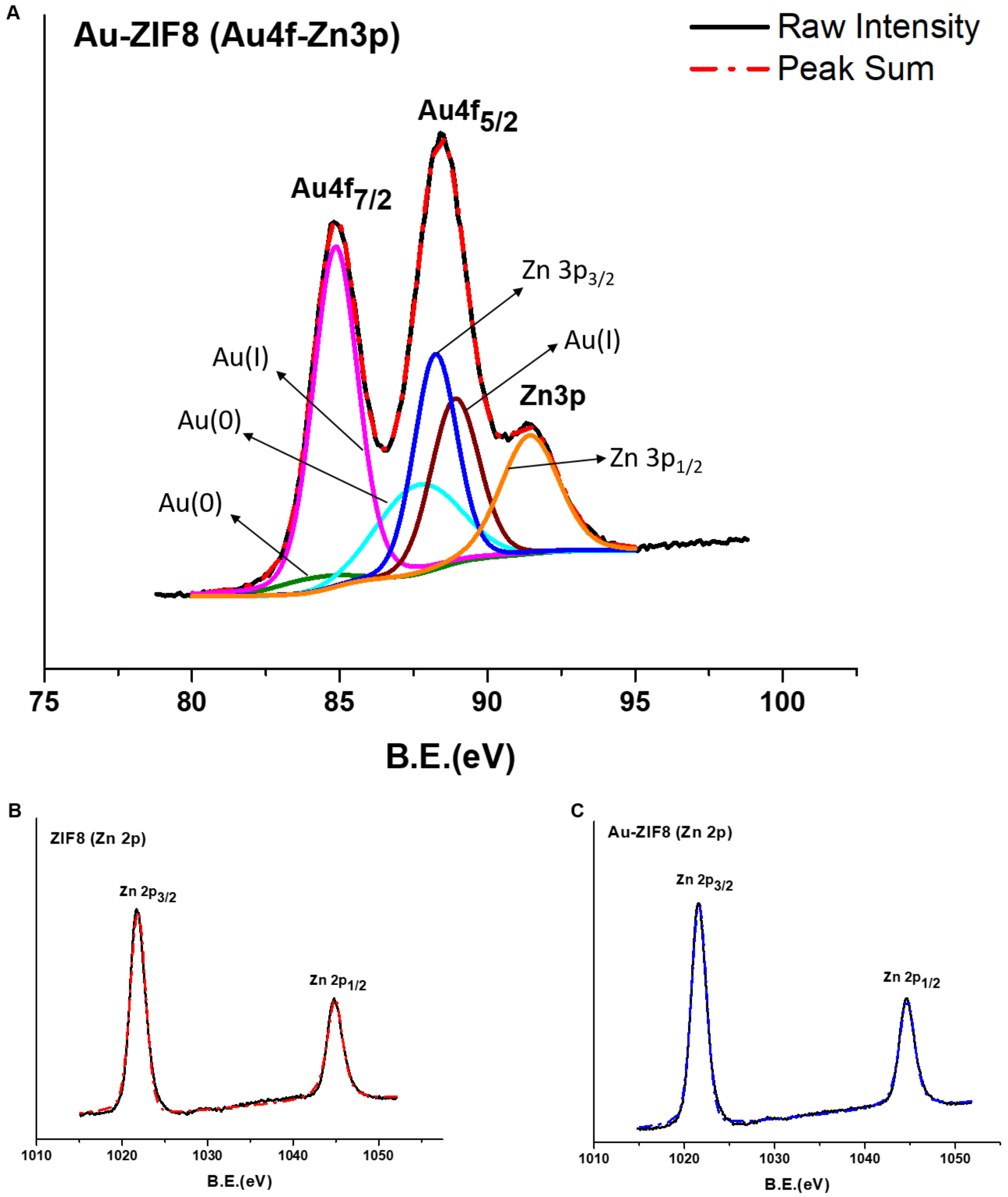


**Figure S3: XPS high resolution (a) Au4f & Zn3p spectra of Au-ZIF8; (b) Zn 2p spectra of ZIF-8; (c) Zn 2p spectra of Au-ZIF-8.**

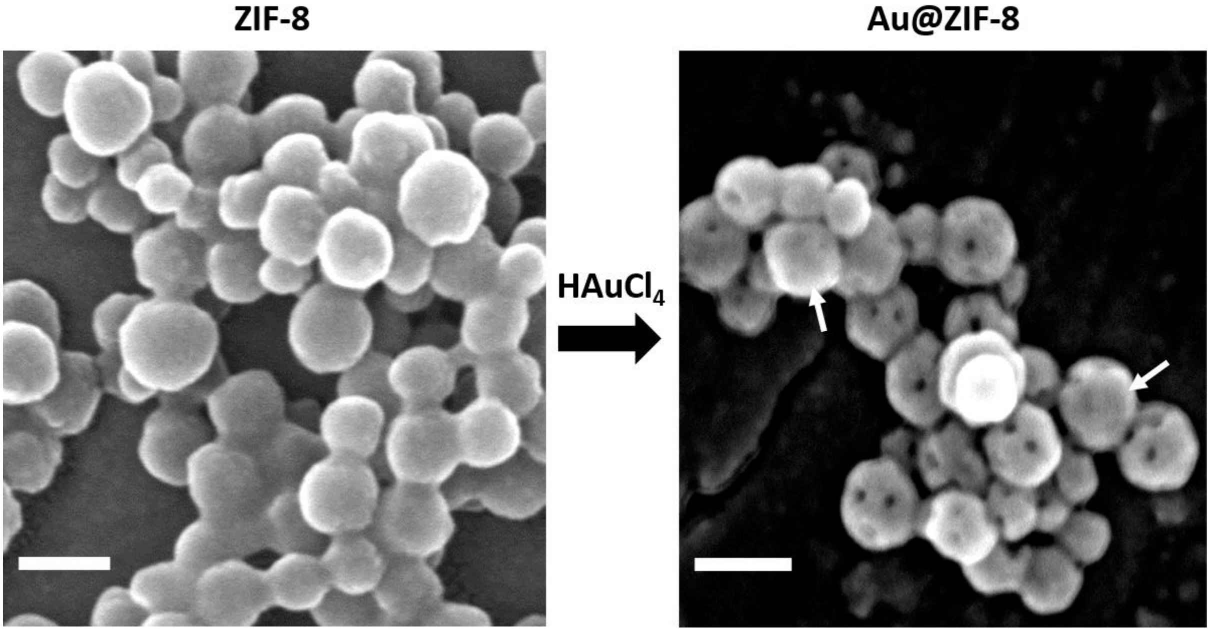


**Figure-S4: Anisotropic & asymmetric etching along three <100> zonal axes of ZIF-8 (2 mg/mL) with chloroauric acid (5 mM). Arrows indicate regions with shallow etching of the three symmetrically opposite <100> axes. (Scale bar – 100 nm)**

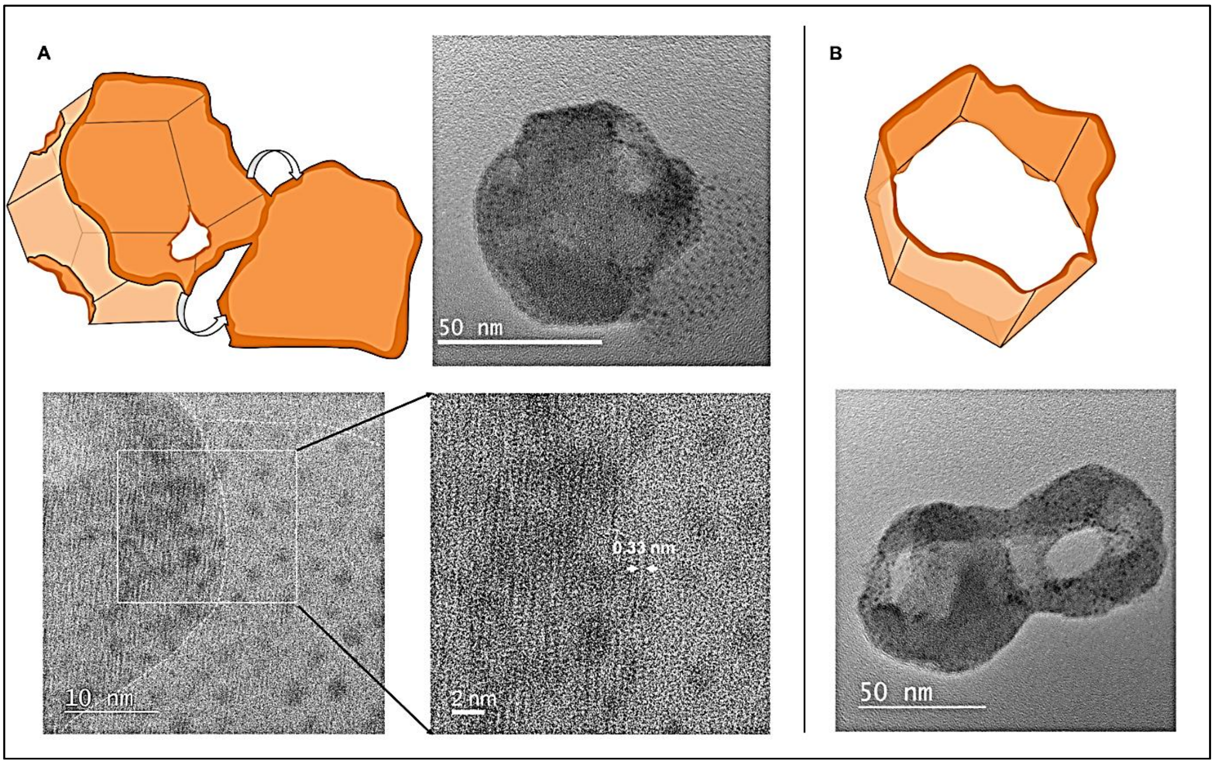


**Figure-S5: (A) Exfoliation of (110) facet from Au@ZIF-8 shell and the high resolution images of the boundary between intact Au@ZIF-8 and the exfoliated layer indicating pore aperture of ~0.33 nm; (B) Morphology of Au@ZIF-8 with symmetrical etching at all six <100> zonal axes.**

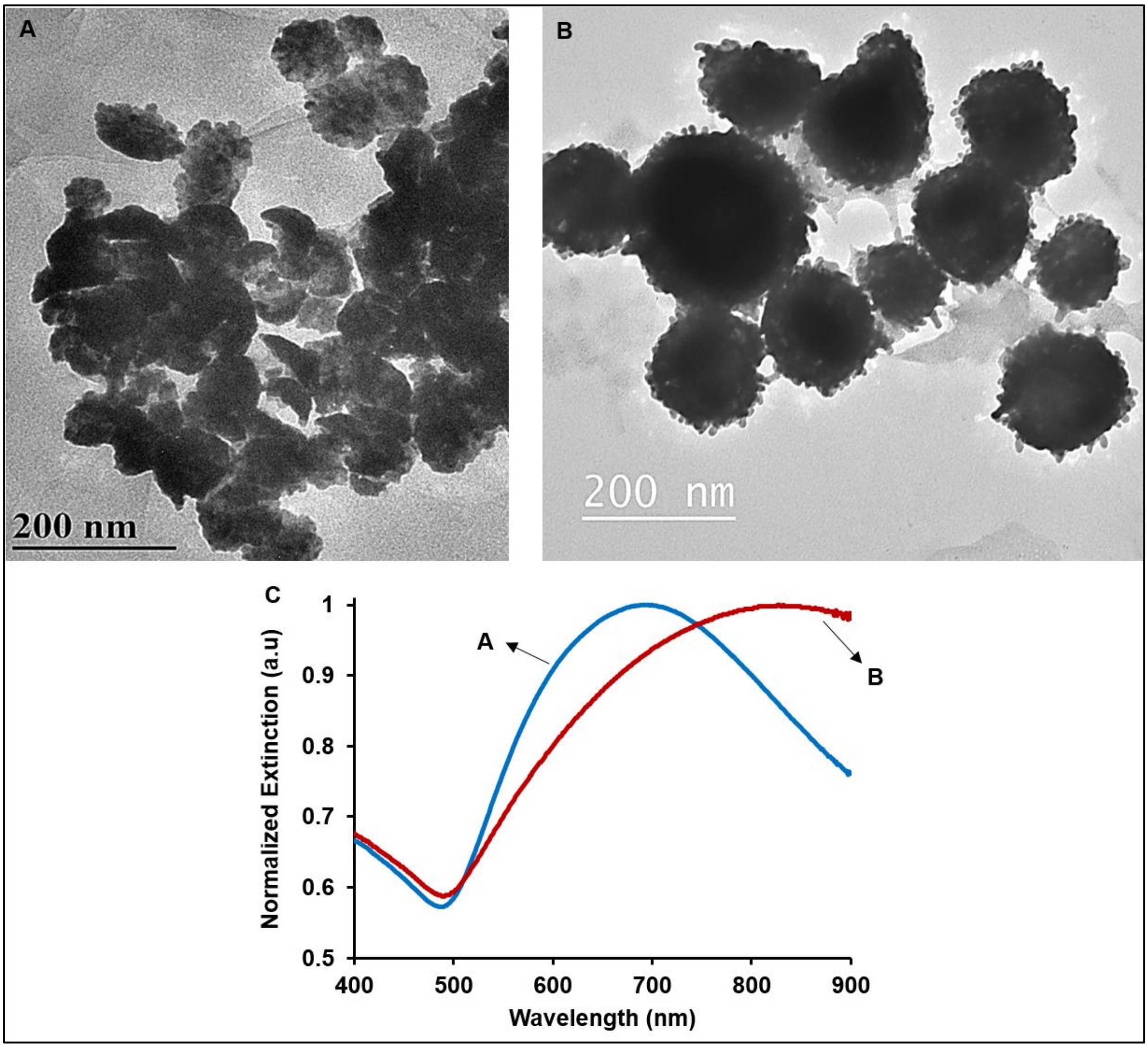


**Figure-S6: TEM images of nanocaps (A) and nano shell (B) prepared from 0.5 mg / mL & 3 mg / mL ZIF-8 respectively and their corresponding extinction spectra (C).**

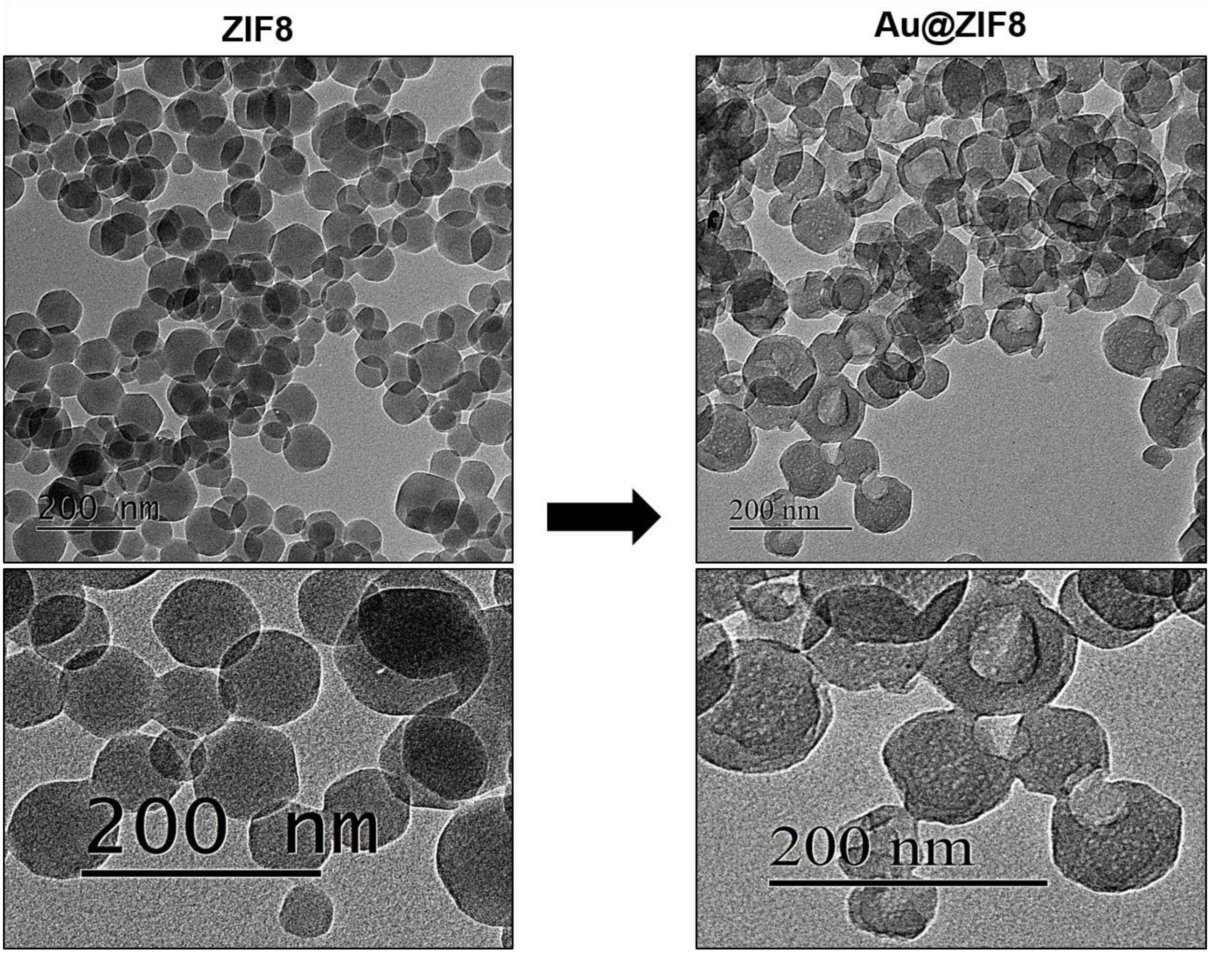


**Figure-S7: TEM images pre (A) and post (B) anisotropic etching of ZIF-8 with chloroauric acid after pre-synthetic adjustment of pH to ~10.**

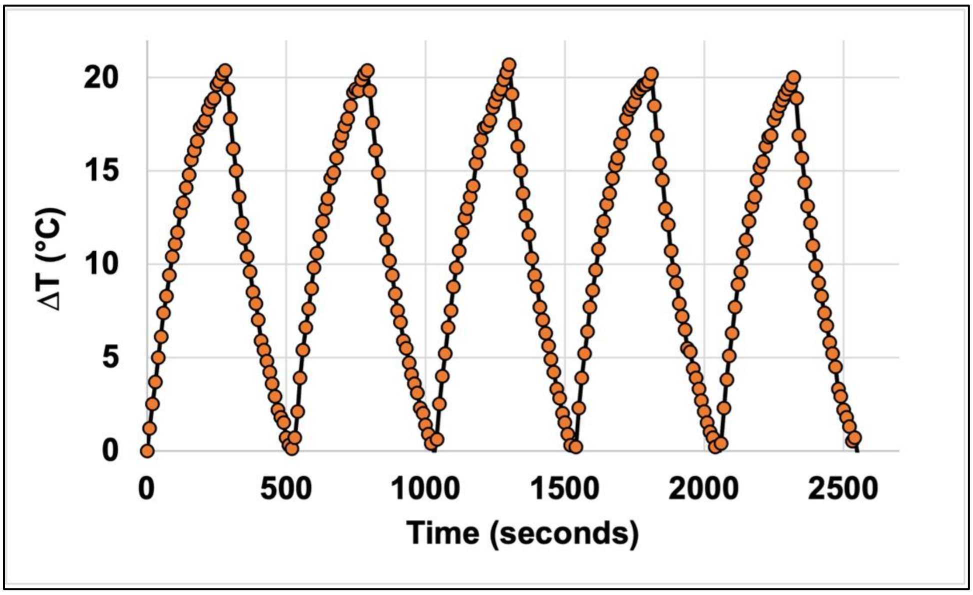


**Figure-S8: Photothermal stability of reconstituted PEGylated SS (1 mg/mL) with 808 nm laser at 1 watt power.**

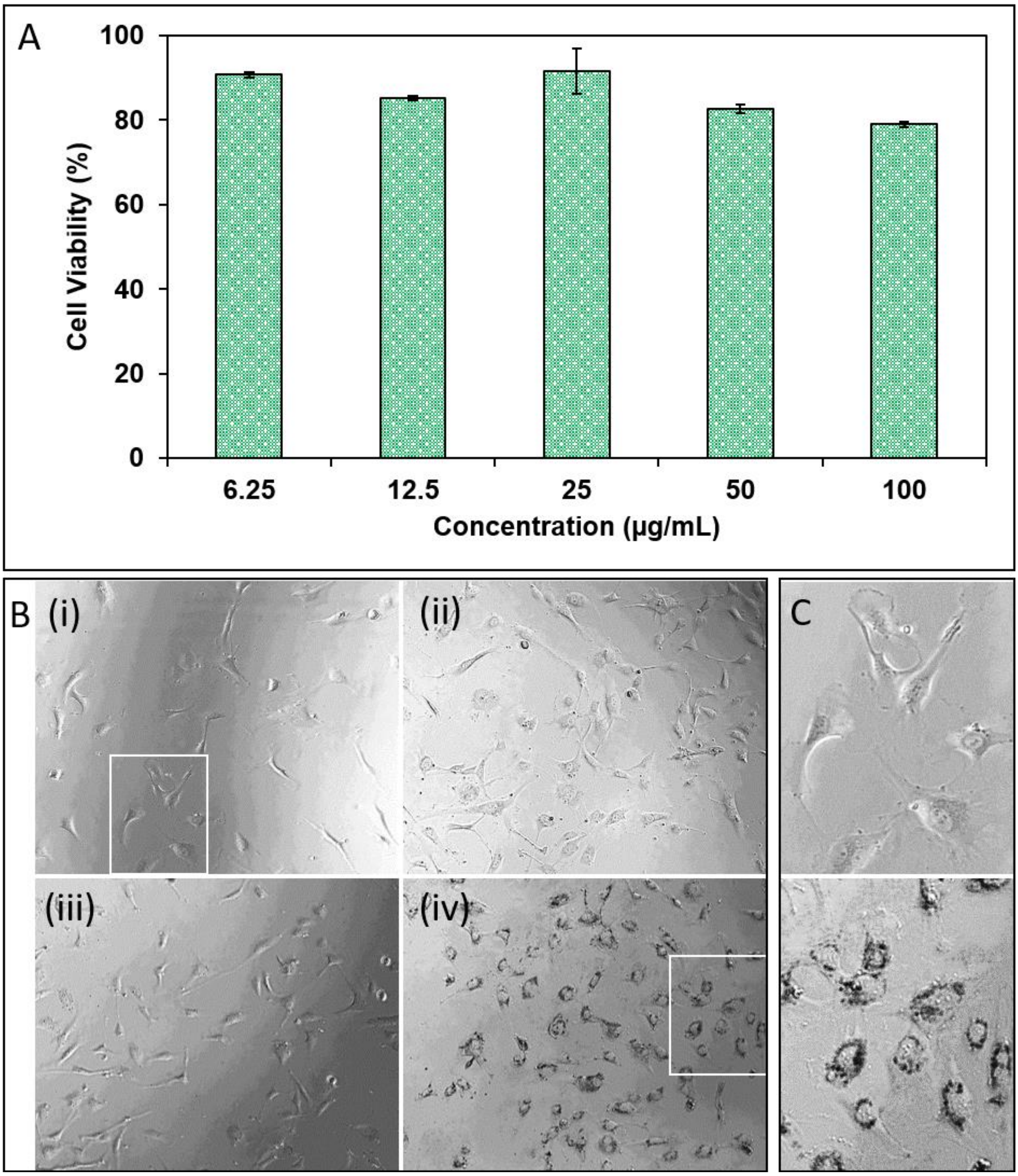


**Figure-S9: (A) Percentage viability of HUVEC cells post treatment with different concentrations of PEGylated SS; (B) Bright field microscopic images of HUVEC cells treated with (i) 0 µg/mL, (ii) 25 µg/mL, (iii) 50 µg/mL and (iv) 100 µg/mL PEGylated SS; (C) Magnified image of the regions highlighted in (i) and (iv).**

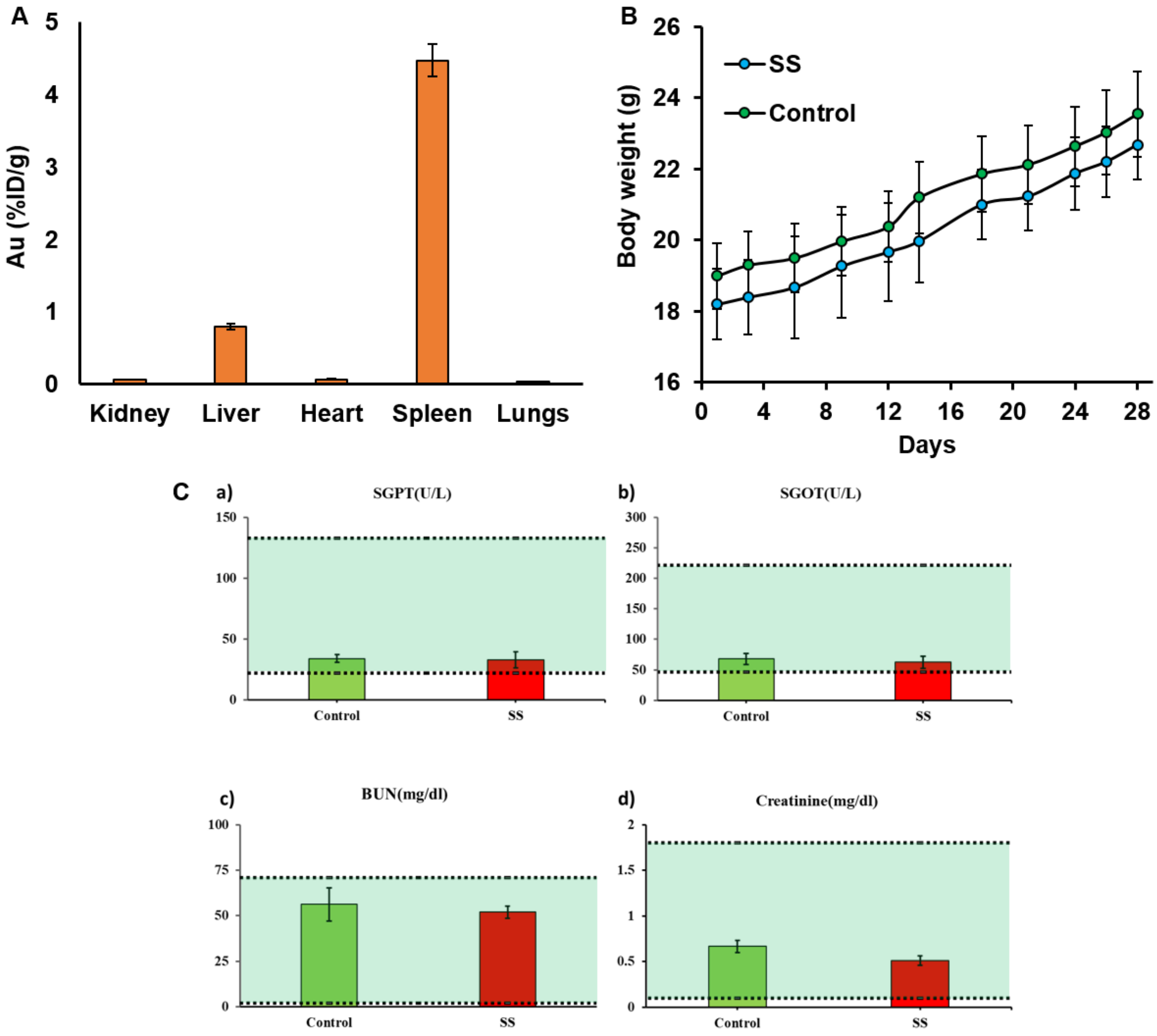


**Figure-S10: (A) *In-vivo* biodistribution of PEGylated SS post intravenous injection at day-28; (B) Body weight (g) of Control and SS injected mice over period of 28 days; (C) Serum biochemical analysis for determining vital organ functioning through a) SGPT b) SGOT c)Blood urea nitrogen (BUN) and d) Creatinine at day-28.**

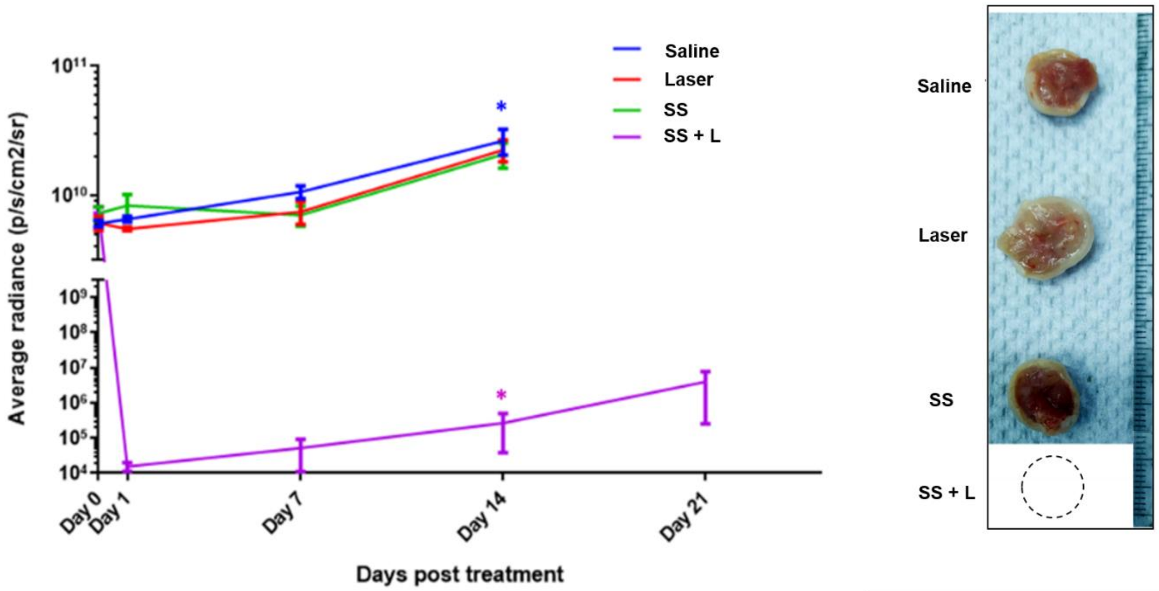


**Figure-S11: Change in bioluminescence signal of 4T1 FL2 tumor grafted on CD1 nude mice post treatment with Saline, Laser, SS and SS+Laser and representative tumors excised from each group post sacrifice (*$p$<0.05).**

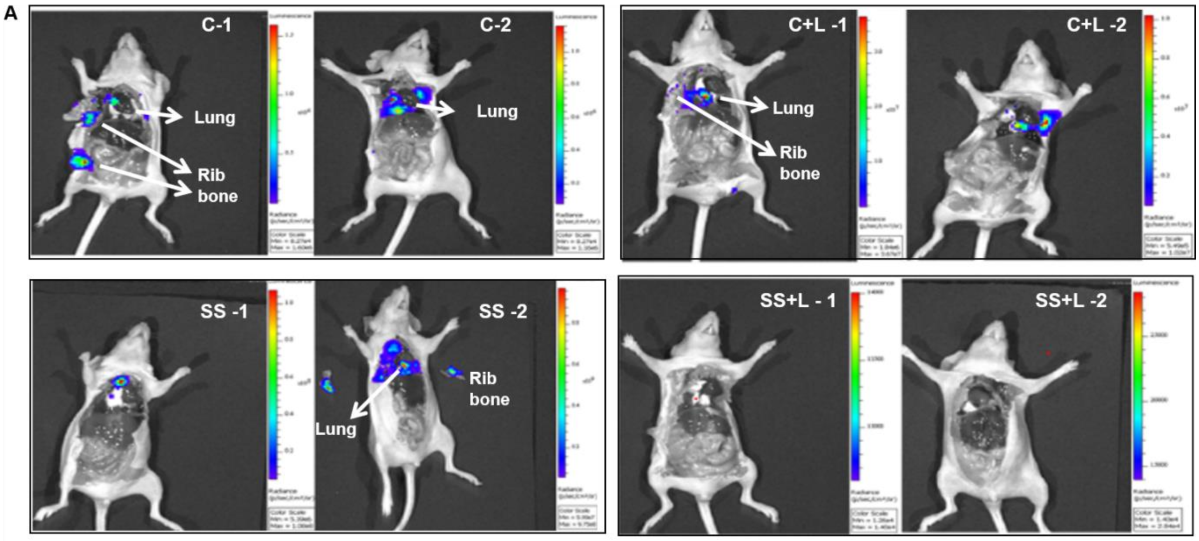


**Figure-S12: (A) Bioluminescence imaging of representative mice from saline (C1 & C2), Saline+Laser (C+L-1 and C+L-2), semi shells (SS-1 & SS-2 and semi shell + Laser (SS+L-1 & SS+L-2) groups with arrows tracing to presenc of tumor in lung and rib bone.**

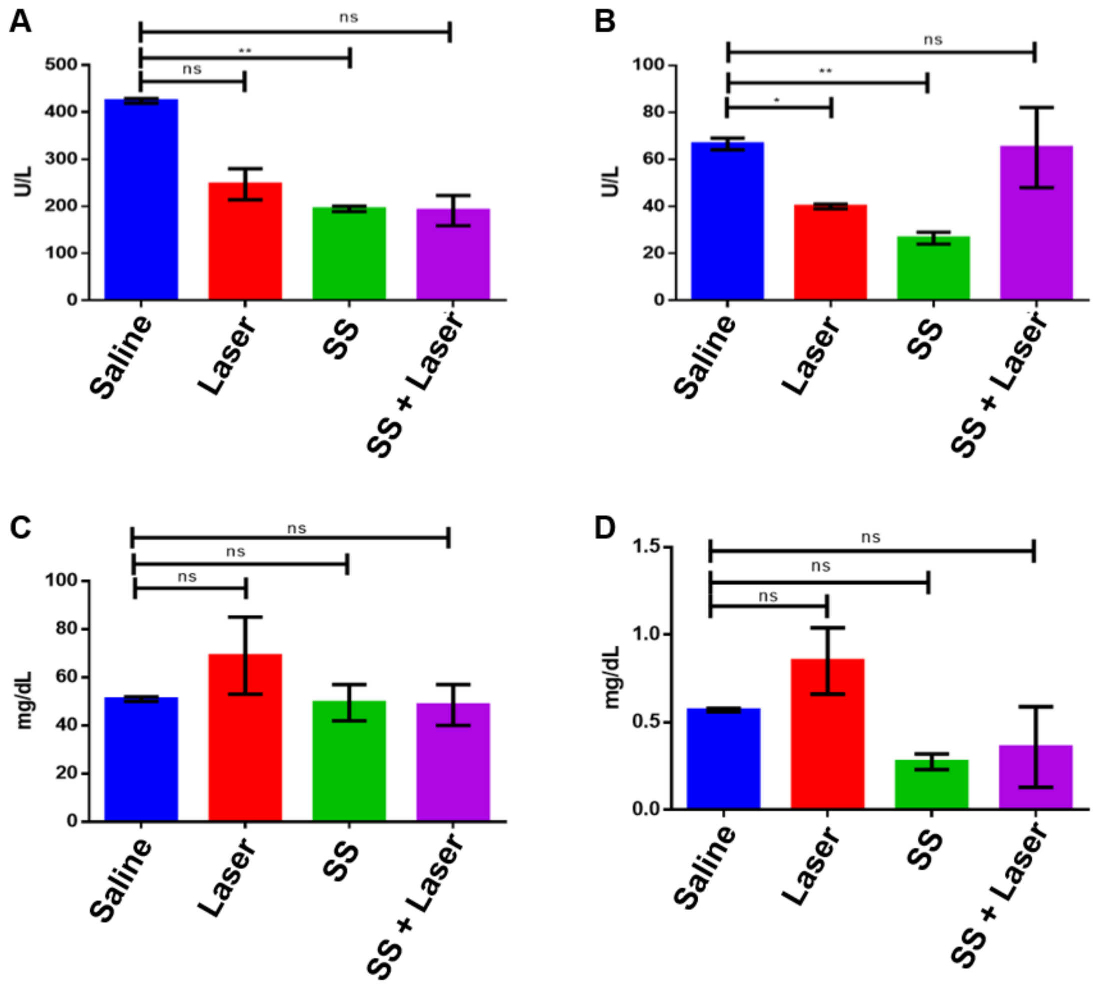


**Figure-S13: Serum biochemical analysis post tumor regression analysis in 4T1 FL2 grafted CD1 nude mice for assessing liver and kidney functioning based on levels of A) SGPT B) SGOT C) BUN and D) Creatinine (ns – non-significant, *$p$<0.05, **$p$<0.01).**

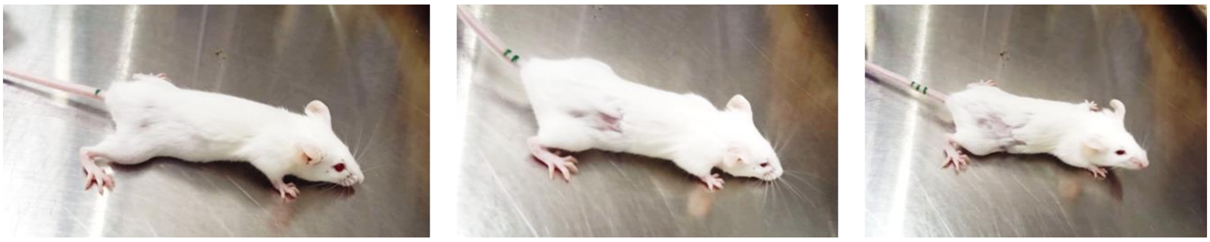

**Figure-S14: Tumor free mice (75% relapse-free survival; n=4) at the end of 3 months post photothermal treatment.**

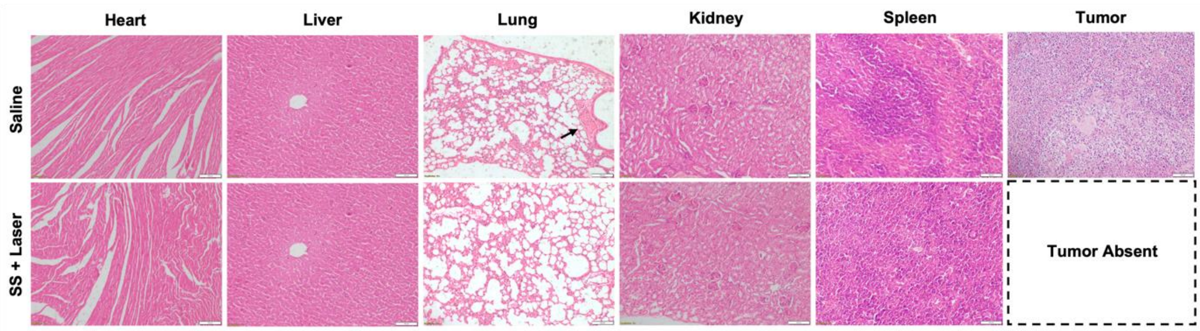


**Figure-S15: A) Microscopic images of haematoxylin & eosin stained sections of vital organs and tumor in saline and SS+Laser treated mice. Black arrow in A indicate metastatic tumor lesion in lung section.**
