# Supplementary material for: Plasmonic semi shells derived from simultaneous in situ gold growth and anisotropic acid etching of ZIF-8 for photothermal ablation of metastatic breast tumor
Source: Commun Chem. 2024 Oct 9;7:231. doi: 10.1038/s42004-024-01317-w (PMC11464763; doi:10.1038/s42004-024-01317-w)
Supplement: Supplementary file 2 — Description of Additional Supplementary Files [file 42004_2024_1317_MOESM2_ESM.pdf]

Description of Additional Supplementary Files

File name: Supplementary Data 1

Description: Source Data

File name: Supplementary Video 1

Description: Reconstitution of Lyophilized SS in 0.9 % (w/v) saline

File name: Supplementary Video 2

Description: Sequential irradiation of freshly reconstituted SS with 750 nm and 808 nm CW lasers at fixed power and time

File name: Supplementary Video 3

Description: On-demand reconstitution of SS in saline for *in vivo* administration

File name: Supplementary Video 4

Description: Thermal imaging of mice treated with SS mediated photothermal therapy
